# Prevalence of depression or depressive symptoms among people living with HIV/AIDS in China: a systematic review and meta-analysis

**DOI:** 10.1186/s12888-018-1741-8

**Published:** 2018-05-31

**Authors:** Tingting Wang, Hanlin Fu, Atipatsa Chiwanda Kaminga, Zhanzhan Li, Guiping Guo, Lizhang Chen, Qiongxuan Li

**Affiliations:** 1 Department of Epidemiology and Health Statistics, Xiangya School of Public Health, Central South University, Changsha, Hunan Province China; 2 Department of Mathematics, Mzuzu University, Mzuzu 2, Malawi; 3 Department of Oncology, Xiangya Hospital, Central South University, Changsha, Hunan Province China; 4 Department of Medical Psychology, The Second Xiangya Hospital of Central South University, Changsha, Hunan Province China

**Keywords:** Prevalence, Depression, Depressive symptoms, HIV/AIDS, Systematic review, Meta-analysis

## Abstract

**Background:**

The number of people living with HIV/AIDS (PLHA) in China continues to increase. Depression, a common mental disorder in this population, may confer a higher likelihood of worse health outcomes. An estimate of the prevalence of this disorder among PLHA is required to guide public health policy, but the published results vary widely and lack accuracy in China. The goal of this study was to estimate the pooled prevalence of depression or depressive symptoms among PLHA in China.

**Methods:**

A systematic literature search of several databases was conducted from inception to June 2017, focusing on studies reporting on depression or depressive symptoms among PLHA in China. The risk of bias of individual studies was assessed using a modified version of the Newcastle-Ottawa scale. The overall prevalence estimates were pooled using random-effects meta-analysis. Differences according to study-level characteristics were examined using stratified meta-analysis and meta-regression.

**Results:**

Seventy-four observational studies including a total of 20,635 PLHA were included. The pooled prevalence of depression or depressive symptoms was 50.8% (95% CI: 46.0–55.5%) among general PLHA, 43.9% (95% CI: 36.2–51.9%) among HIV-positive men who have sex with men, 85.6% (95% CI: 64.1–95.2%) among HIV-positive former blood/plasma donors, and 51.6% (95% CI: 31.9–70.8%) among other HIV-positive populations. Significant heterogeneity was detected across studies regarding these prevalence estimates. Heterogeneity in the prevalence of depression among the general population of PLHA was partially explained by the geographic location and baseline survey year.

**Conclusions:**

Because of the significant heterogeneity detected across studies regarding these prevalence estimates of depression or depressive symptoms, the results must be interpreted with caution. Our findings suggest that the estimates of depression or depressive symptoms among PLHA in China are considerable, which highlights the need to integrate screening and providing treatment for mental disorders in the treatment package offered to PLHA, which would ultimately lead to better health outcomes in PLHA.

**Electronic supplementary material:**

The online version of this article (10.1186/s12888-018-1741-8) contains supplementary material, which is available to authorized users.

## Background

Human immunodeficiency virus (HIV) infection remains a significant social issue worldwide. Estimates reported by the World Health Organization (WHO) suggested that 36.7 million people were living with HIV infection and acquired immune deficiency syndrome (AIDS) at the end of 2015, with 2.1 million new infections and 1.1 million deaths due to HIV-related causes. Most people living with HIV/AIDS (PLHA) are in low-income and middle-income countries [1]. As a middle-income country, the number of PLHA continues to increase in China, although the nationwide epidemic situation of HIV/AIDS remains at a low rate. According to the Chinese Center for Disease Control and Prevention, there were 0.50 million people infected with HIV at the end of 2014 in China [2], and in June 2017, this number increased to 0.66 million, 41.7% of whom were AIDS patients [3]. From 2014 to 2016, more than 10,000 people were infected with HIV every year [2–4].

Since the introduction of highly active antiretroviral therapy (ART) in the late 1990s, a large percentage of individuals with HIV-infection have been able to avoid death and live longer in a healthy condition. Nevertheless, due to social stigma, sexual dysfunction, long-term physical discomfort and illness, side effects of antiretroviral therapy, and neurobiological changes [5, 6], PLHA are at a higher risk of mental disorders, particularly depression. Evidence suggests that, depression occurs more commonly in HIV-positive individuals, with a prevalence that is two to four times higher compared with comparable HIV-negative individuals or the general population [7–9]. Individuals with HIV infection and depression perform more poorly on clinical outcomes [10]. In fact, evidence suggests that depression may reduce antiretroviral therapy adherence and quality of life, weaken the physical function and therapeutic effect [11, 12], and confer a higher rate of medical comorbidities [13, 14]. Moreover, in several studies, depression has been found to be associated with higher HIV viral loads and lower CD4 counts, even after controlling for the effects of adherence, which predict a worsening disease progression and mortality [11, 15–20]. Even depressive symptoms, which do not necessarily meet the entire diagnostic criteria for a depressive disorder, have been identified as a significant factor associated with worse health outcomes among people with HIV infection, including impaired immunological response and mortality [21–27]. Therefore, screening for depression or depressive symptoms is an overriding concern in identifying significant risk factors for health outcomes among those who are living with HIV/AIDS.

Given the importance of the association between HIV infection and depression, scholars have been committed to the epidemiological study of depression in China. While revealing a high occurrence of depression or depressive symptoms among PLHA, the results of existing studies have been fragmentary and inconsistent. For example, the prevalence among PLHA in Changsha City was 18.3% [28], whereas among PLHA in Wuhan City, it was 40.4% [29], among PLHA in Shanghai City, it was 60.3% [29], and among PLHA in Kunming City, it was 81.5% [30]. This knowledge gap is an obstacle to policy and practice. For example, the success of a screening program is sensitive to base prevalence. As the inconsistencies are outstanding in the current literature, it would be useful to analyze the data provided in the scientific literature using integrated approaches to establish the extent of depression or depressive symptoms among PLHA and clarify the reasons for the differences.

Therefore, in the present study, the objective was to conduct a systematic review and meta-analysis of studies to determine the prevalence of depression or depressive symptoms among PLHA in China and to explore the possible causes of the inconsistencies in the current estimates.

## Methods

### Search strategy

Two reviewers independently searched the EMBASE, Web of Science, PubMed, Wanfang, China Biology Medicine disc, China National Knowledge Infrastructure, and Weipu databases from inception to June 2017 for articles in English and Chinese, with no restriction on the year of the study.. The following search terms were used: human immunodeficiency virus, acquired immune deficiency syndrome, HIV, AIDS, depression, depressive disorder, depressive symptom, mental disorder, mental health, mood disorder, affective disorder, psychological health, and psychiatric. Search strategy details are shown in Additional file 1. In addition, the reviewers manually searched the reference lists of identified articles to identify any relevant studies missed in the initial search.

### Study selection

At the stage of titles and abstracts screening, we purposely broadened the inclusion criteria to obtain any relevant study. First, studies were considered for inclusion if they were published in Chinese or English and reported on depression or depressive symptoms among PLHA. Then, the full texts of all selected studies were reviewed. Articles were included if they 1) were cross-sectional or cohort in design, 2) reported PLHA in China as a primary study population, 3) used a standard instrument to assess for depression or depressive symptoms, and 4) provided information about prevalence estimate of depression or depressive symptoms among PLHA. Conversely, articles were excluded if they 1) were review papers, conference abstracts, case reports, experimental studies, qualitative studies or case-control studies, 2) had incomplete or unclear data, or 3) were duplicate publications. Studies using only the data obtained from the National Health Insurance Research Database (NHIRD) were also excluded because of the possibility of underestimation. When there was more than one study involving the same population of PLHA, only the most recent published or comprehensive one was included. In addition, if the same data were published in both Chinese and English, then the articles published in Chinese were excluded.

### Data extraction

Two reviewers independently extracted and evaluated the data for each included article using a self-designed data abstraction form. Disagreements were resolved through discussion or consultation with a third reviewer when consensus could not be achieved. The following data were extracted: the first author, year of publication, duration of data collection, geographic location, study design, sample source, subjects, sample size, average age of participants (mean or median), number and percentage of male participants, screening or diagnostic method, outcome definition (screening instrument cutoff or diagnostic criteria) and reported prevalence estimates of depression or depressive symptoms among PLHA. If a study reported more than one estimate assessed by different measurement tool, the one detected by the more valid measurement tool (i.e., the tool with higher specificity and sensitivity) was extracted. When there were multiple estimates over time in the same sample of a study, the first one was chosen.

### Assessment of risk of bias

The risk of bias in the included studies was assessed using a modified version of the Newcastle-Ottawa scale (NOS) which was referred to the version used in the meta-analysis conducted by Rotenstein et al. to estimate the prevalence of depression or depressive symptoms in medical students [31]. The tool contained five items, which determine the risk of bias, including sample representativeness, sample size, response rate, ascertainment of depression, and quality of descriptive statistics reporting (for details, see Additional file 2). The five criteria were assessed as either “1 point” or “0 point”. The higher the score, the lower the risk of bias in an individual study. According to Rotenstein et al. [31], a study was rated as having a high risk of bias if less than 3 points were given, and a low risk of bias if 3 or more points were given.

### Statistical analysis

All analyses were performed using R version 3.4.1 (R Foundation for Statistical Computing), ‘meta’ package (version 4.8–4). In the presence of between-study heterogeneity, the pooled prevalence estimates and corresponding 95% confidence intervals (CIs) were calculated using random-effects meta-analyses. Data from studies based on HIV-positive sub-populations with specific characteristics (i.e., men who have sex with men [MSM], pregnant women, tuberculosis [TB] patients, injected drug users [IDUs] and former blood/plasma donors [FBPD]) were analyzed separately when at least six studies were available. As fewer than six studies reported data on HIV-positive pregnant women, HIV-TB co-infected individuals and HIV-positive IDUs, studies on those sub-populations were combined as “other HIV-positive population” to estimate the pooled prevalence. Cochran Q test and the I^2^ statistic were used to assess the between-study heterogeneity. The Cochran Q test was used to evaluate whether the variation across studies was compatible with chance, and *p* <  0.1 was considered to indicate significant heterogeneity. The I^2^ statistic was a quantitative indicator used to evaluate the percentage of total variance in prevalence estimates due to statistical heterogeneity rather than chance, or sampling error (I^2^ > 75% indicates high heterogeneity, 51–75% indicates substantial heterogeneity, 26–50% indicates moderate heterogeneity, and ≤ 25% indicates low heterogeneity).

Results from included studies were grouped according to pre-specified study-level characteristics, and then they were compared using subgroup meta-analysis (for screening instrument cut-off or diagnostic criteria, geographic location, sample source and total NOS score) or random-effects meta-regression (for baseline survey year, sample size, age and sex). The difference between subgroups was examined using the Cochran Q test (*p* <  0.05 indicated statistically significant differences). To determine the influence of individual studies on the pooled prevalence estimates, sensitivity analyses were performed by serially repeating the meta-analysis after the exclusion of each included study. If the point estimate of the new pooled prevalence is outside of the 95% confidence interval of the original pooled prevalence, it can be determined that the study which has been excluded to get the new prevalence has an significant effect on the original pooled prevalence. Publication bias was evaluated using Egger’s line regression test (*p* <  0.05 indicated statistically significant differences). Preferred Reporting Items for Systematic Reviews and Meta-analysis guidelines were strictly adhered to wherever appropriate [32].

## Results

### Identification and characteristics of studies

**Fig. 1:**
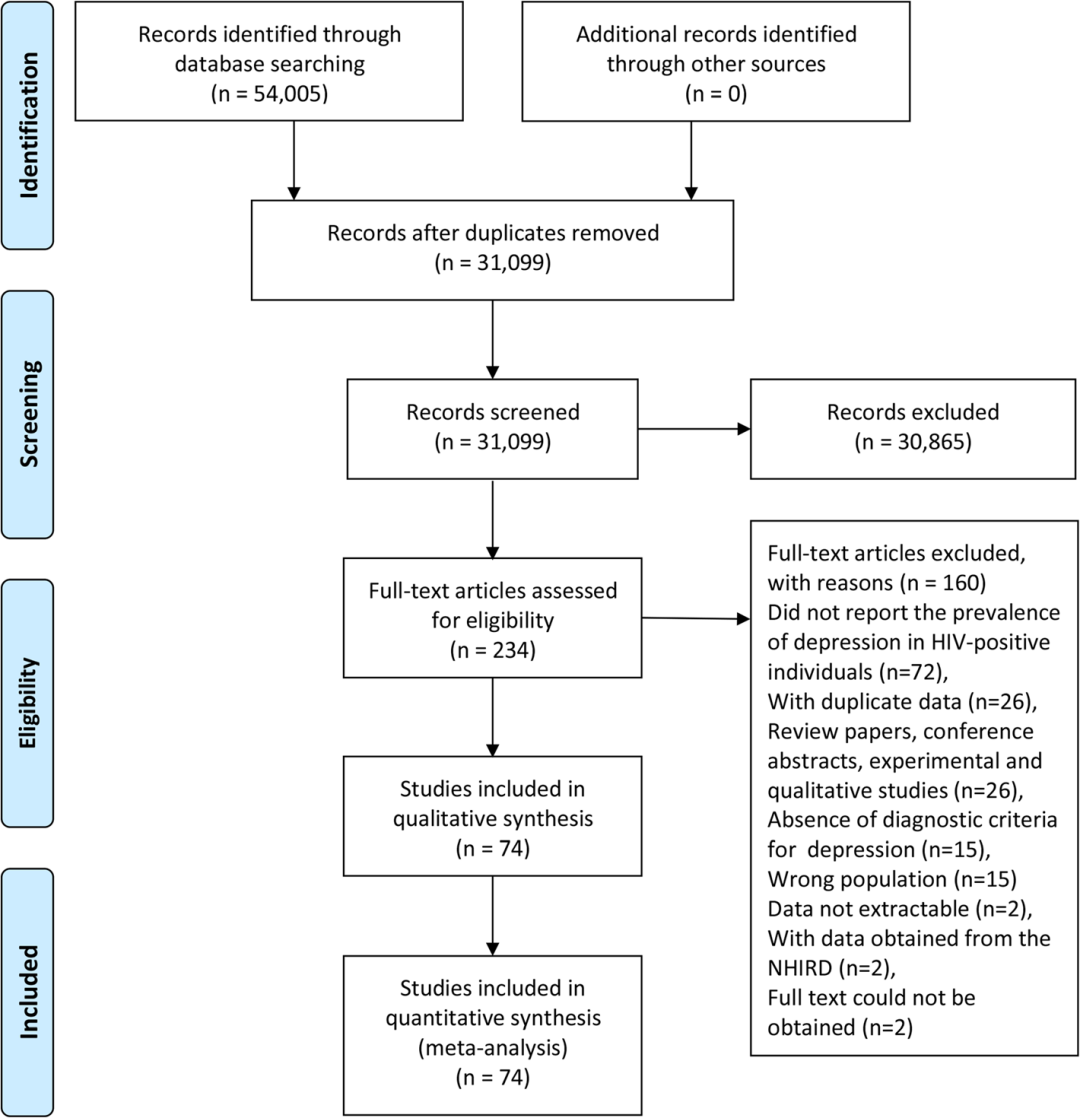
Flow diagram of included/excluded studies

In total, 54,005 unique citations were identified after an initial search, 53,771 of which were excluded after removing duplicate papers and screening titles and abstracts (Fig. 1). Then, the full text of 234 articles were reviewed, 74 of which [9, 28–30, 33–102] were considered to be eligible and included in the systematic review and meta-analysis.

In the 74 studies, there were a total of 20,635 PLHA. The median number of participants in those studies was 185 (range: 28 to 4103). Sixty-seven studies were conducted in one of the seven areas (twenty-one in East China, seventeen in Central China, ten in South China, nine in Southwest China, six in North China, three in Northeast China and one in Northwest China), six studies were conducted in two or more areas and one study did not report the study site. The papers were published between 2004 and 2017, and more than 70% (54/74 studies) were published between 2011 and 2017. Seventy-one cross-sectional studies (*n* = 20,154) and three longitudinal studies (*n* = 481) reported on the prevalence of depression or depressive symptoms, and twenty-three of the seventy-three observational studies focused on specific sub-populations (ten on MSM, seven on FBPD, two on pregnant women, two on HIV-TB co-infected individuals, and two on IDUs). More details are shown in an additional table file (see Additional file 3).

### Study quality

Modified NOS score components for all 74 individual studies are shown in Additional file 4 and Additional file 5. Fifty-seven studies (77.0%) had an overall rating of low risk, while the rest were rated as high. One-fifth of the studies scored 1 point on each of these five items. The overall sample representativeness was fair, as more than half of the studies (41, 55.4%) sampled PLHA from HIV-infected individuals databases of the provincial or municipal Center for Disease Control and Prevention or from multiple study sites. Forty-one studies reported responses of at least 70%, and of these more than 90% sampled 100 or more PLHA.

### Depression or depressive symptoms among the general PLHA

#### Estimate of overall prevalence of depression or depressive symptoms among the general population of PLHA

**Fig. 2:**
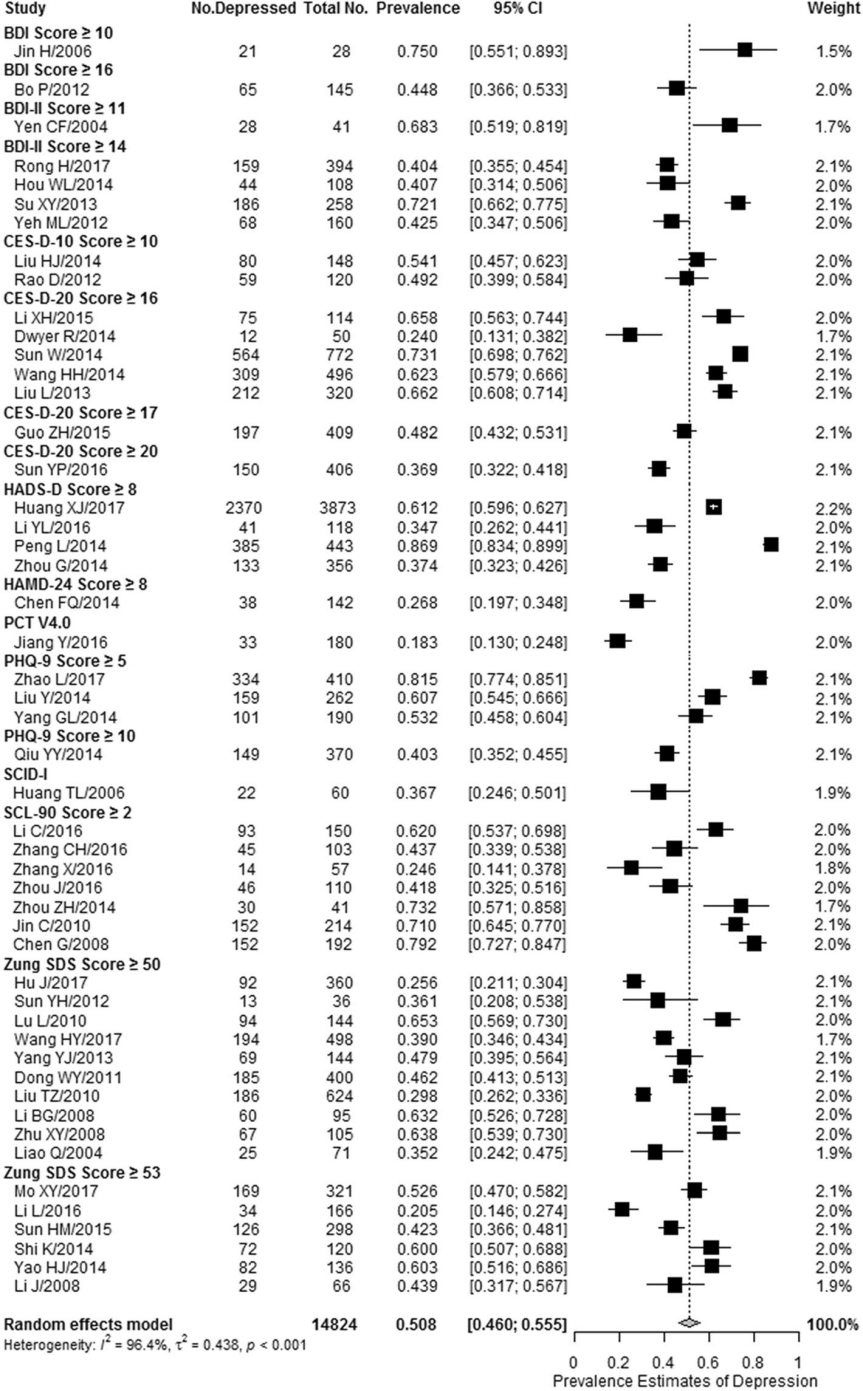
Forest plot of the prevalence of depression or depressive symptoms among the general people living with HIV/AIDS in China. The vertical dotted line indicates the overall effect size of all studies combined. The studies are ordered alphabetically by screening instrument and cutoff score, and then sorted by decreasing publication year within each instrument. BDI, Beck Depression Inventory; CES-D-10, 10-item Center for Epidemiological Studies Depression Scale; CES-D-20, 20-item Center for Epidemiological Studies Depression Scale; HADS-D, Hospital Anxiety and Depression Scale; HAMD-24, 24-item Hamilton Depression Rating Scale; PCT V4.0, Psychological ‘Computerized Tomography’ 4.0 Vision; PHQ-9, 9-item Patient Health Questionnaire; SCID-I, Structured Clinical Interview for the fourth edition of the Diagnostic and Statistical Manual for Mental Disorders Axis I Disorders; SCL-90, 90-item Symptom Check List; Zung-SDS, Zung Self-Rating Depression Scale

The prevalence estimates of depression or depressive symptoms among the general PLHA reported by 50 included studies ranged from 18.3 to 86.9%. Meta-analytic pooling of these prevalence estimates yielded a crude summary prevalence of 50.8% (8023/14,824 individuals, 95% CI: 46.0–55.5%), with significant between-study heterogeneity present (I^2^ = 96.4%, *p* <  0.001) (Fig. 2). No evidence of publication bias was detected using the Egger’s test (*t* = − 1.549, *p* = 0.128). Sensitivity analysis showed that none of the studies had a significant influence on the pooled prevalence estimate (see Additional file 6).

To further characterize the range of prevalence estimates of depression or depressive symptoms, a stratified analysis was conducted, based on the screening instruments and cut-off scores used in these methodologically diverse studies (Table 1). Summary prevalence estimates of depression or depressive symptoms ranged from 18.3% (95% CI: 13.0–24.8%) for Psychological “Computerized Tomography” 4.0 Vision (PCT V4.0) to 75.0% (95% CI: 55.1–89.3%) for the Beck Depression Inventory (BDI), with a cut-off score of 10 or greater. The median summary prevalence estimate was 48.2% (95% CI: 43.2–53.1%) for the 20-item Center for Epidemiological Studies Depression Scale (CES-D-20), with a cut-off score of 17 or greater.

**Table 1:** Meta-analyses of the prevalence of depression or depressive symptoms among the general PLHA in China stratified by instrument and cutoff score

Screening instrument and cutoff score	No. of Studies	No. Depressed	Total No.	Prevalence, % (95% CI)	I^2^ (%)	*P* value for heterogeneity
Beck Depression Inventory Score ≥ 10	1	21	28	75.0 (55.1, 89.3)	–	–
Beck Depression Inventory Score ≥ 16	1	65	145	44.8 (36.6, 53.3)	–	–
Beck Depression Inventory II Score ≥ 11	1	28	41	68.3 (51.9, 81.9)	–	–
Beck Depression Inventory II Score ≥ 14	4	457	920	49.3 (33.2, 65.5)	95.6	< 0.001
10-item Center for Epidemiologic Studies Depression Scale Score ≥ 10	2	139	268	51.9 (45.9, 57.8)	0	0.426
20-item Center for Epidemiologic Studies Depression Scale Score ≥ 16	5	1172	1752	60.9 (51.2, 69.8)	91.9	< 0.001
20-item Center for Epidemiologic Studies Depression Scale Score ≥ 17	1	197	409	48.2 (43.2, 53.1)	–	–
20-item Center for Epidemiologic Studies Depression Scale Score ≥ 20	1	150	406	36.9 (32.2, 41.8)	–	–
Hospital Anxiety and Depression Scale Score ≥ 8	4	2929	4790	57.6 (36.7, 76.0)	98.6	< 0.001
24-item Hamilton Depression Rating Scale Score ≥ 8	1	38	142	26.8 (20.1, 34.6)	–	–
Psychological “Computerized Tomography”4.0 Vision	1	33	180	18.3 (13.0, 24.8)	–	–
9-item Patient Health Questionnaire Score ≥ 5	3	594	862	66.4 (46.9, 81.6)	96.5	< 0.001
Patient Health Questionnaire-9 Score ≥ 10	1	149	370	40.3 (35.2, 45.5)	–	–
Structured Clinical Interview for the fourth edition of the Diagnostic and Statistical Manual for Mental Disorders Axis I Disorders	1	22	60	36.7 (24.6, 50.1)	–	–
90-item Symptom Check List Score ≥ 2	7	532	867	57.4 (42.9, 70.7)	93.4	< 0.001
Zung Self-Rating Depression Scale Score ≥ 50	10	985	2477	44.8 (36.3, 53.6)	93.8	< 0.001
Zung Self-Rating Depression Scale Score ≥ 53	6	512	1107	46.0 (35.0, 57.5)	92.4	< 0.001

*PLHA* people living with HIV/AIDS

### Subgroup analysis and meta-regression

Statistically significant differences in prevalence estimates were identified among studies conducted in different areas (Q = 41.3, *p* <  0.001). When stratified by the sample source, the pooled prevalence estimates among the PLHA from the 20 community-based samples (55.3, 95% CI: 47.0–63.4%) was comparable to the PLHA from the 30 studies reporting on hospital-based samples (47.6, 95% CI: 41.7–53.6%) (Q = 2.2, *p* = 0.141). Similarly, there were no significant differences in the prevalence estimates of depression or depressive symptom between studies with total NOS score < 3 points and studies with total ≥ 3 points (Q = 2.5, *p* = 0.117). Data are shown in Table 2.

**Table 2:** Meta-analyses of the prevalence of depression or depressive symptoms among PLHA in China stratified by study-level characteristics

Characteristics	No. of Studies	No. Depressed	Total No.	Prevalence, % (95% CI)	I^2^ (%)	*P* value for heterogeneity	Test for subgroup differences	
							Q (df)	*P* value
Geographic location							41.3 (7)	< 0.001
Central China	13	1836	3927	48.8 (40.3, 57.5)	96.3	< 0.001		
Cross-region	4	2649	4279	65.7 (58.3, 72.4)	78.8	0.003		
East China	14	927	2165	47.4 (38.5, 56.5)	93.3	< 0.001		
North China	4	126	345	33.5 (22.9, 46.0)	79.9	0.002		
Northeast	3	869	1242	67.8 (60.9, 74.0)	80.1	0.007		
Northwest	1	45	103	43.7 (33.9, 53.8)	–	–		
South China	7	1019	1831	52.0 (37.2, 66.5)	97.1	< 0.001		
Southwest	4	552	932	55.7 (29.1, 79.4)	98.1	< 0.001		
Sample source							2.2 (1)	0.141
Community-based	20	2773	4840	55.3 (47.0, 63.4)	96.5	< 0.001		
Hospital-based	30	5250	9984	47.6 (41.7, 53.6)	96.3	< 0.001		
Total score							2.5 (1)	0.117
< 3 points	12	504	1167	43.8 (34.4, 53.8)	90.2	< 0.001		
≥ 3 points	38	7519	13,657	52.9 (47.5, 58.2)	96.9	< 0.001		

PLHA people living with HIV/AIDS

The results of the random-effects meta-regression showed that the prevalence estimates of depression or depressive symptoms significantly varied with the baseline survey year (slope = − 8.3% per 1-year increase [95% CI: -14.2% to − 2.4%]; Q = 7.5, *p* = 0.006). but did not significantly vary with the sample size (slope = 1.5% per 100-individual increase [95% CI: -2.1 to 5.0%]; Q = 0.7, *p* = 0.418), mean or median age (slope = 3.9% per 1-year increase [95% CI: -0.1 to 8.8%]; Q = 2.5, *p* = 0.115), sex (slope = − 0.7% per percentage increase in male individuals [95% CI: -2.0 to 0.7%]; Q = 1.0, *p* = 0.323) or antiretroviral therapy (ART) (slope = − 0.1% per percentage increase in individuals with ART [95% CI: -0.9 to 0.6%]; Q = 0.1, *p* = 0.740).

#### Heterogeneity within the depression survey instruments

To identify potential sources of heterogeneity independent of assessment method, stratified meta-analysis and univariate meta-regression analysis were conducted within subgroups of studies using the same instruments when at least five studies were available. An additional file shows this process in more detail (see Additional file 7). No significant differences were observed between community-based and hospital-based studies, as well as studies with total NOS score < 3 points and ≥ 3 points, within any instruments. Heterogeneity was partially accounted for by geographic location, as studies conducted in North China yielded lower depression or depressive symptoms prevalence estimates than studies conducted in Central China (24.0% [95% CI: 14.2–37.7%] vs 62.9% [95% CI: 59.0–66.7%]), as well as studies conducted in Central China (24.0% [95% CI: 14.2–37.7%] vs 70.0% [95% CI: 63.0–76.2%]) among five studies using the CES-D-20 with a cutoff score of 16 or greater (see Additional file 7 Table S1).

The baseline survey year significantly contributed to the observed notable heterogeneity among the studies using the Zung Self-Rating Depression Scale (Zung SDS), with a cut-off score of 50 or greater, and the 90-item Symptom Checklist (SCL-90), with a cut-off score of 2 or greater. Similarly, age also accounted for between-study heterogeneity within two instruments, Zung SDS score ≥ 50 and CES-D-20 score ≥ 16. Sample size also significantly contributed to the observed notable heterogeneity within three instruments (Zung SDS score ≥ 50, SCL-90 score ≥ 2 and CES-D-20 score ≥ 16), although the results were inconsistent (i.e., two analyses suggested that the prevalence estimate of depression was increasing with sample size, while a third one suggested that it was decreasing). Sex and ART did not significantly contribute to the between-study heterogeneity within any of the four instruments (see Additional file 7 Table S2).

### Depression or depressive symptoms in specific PLHA

**Fig. 3:**
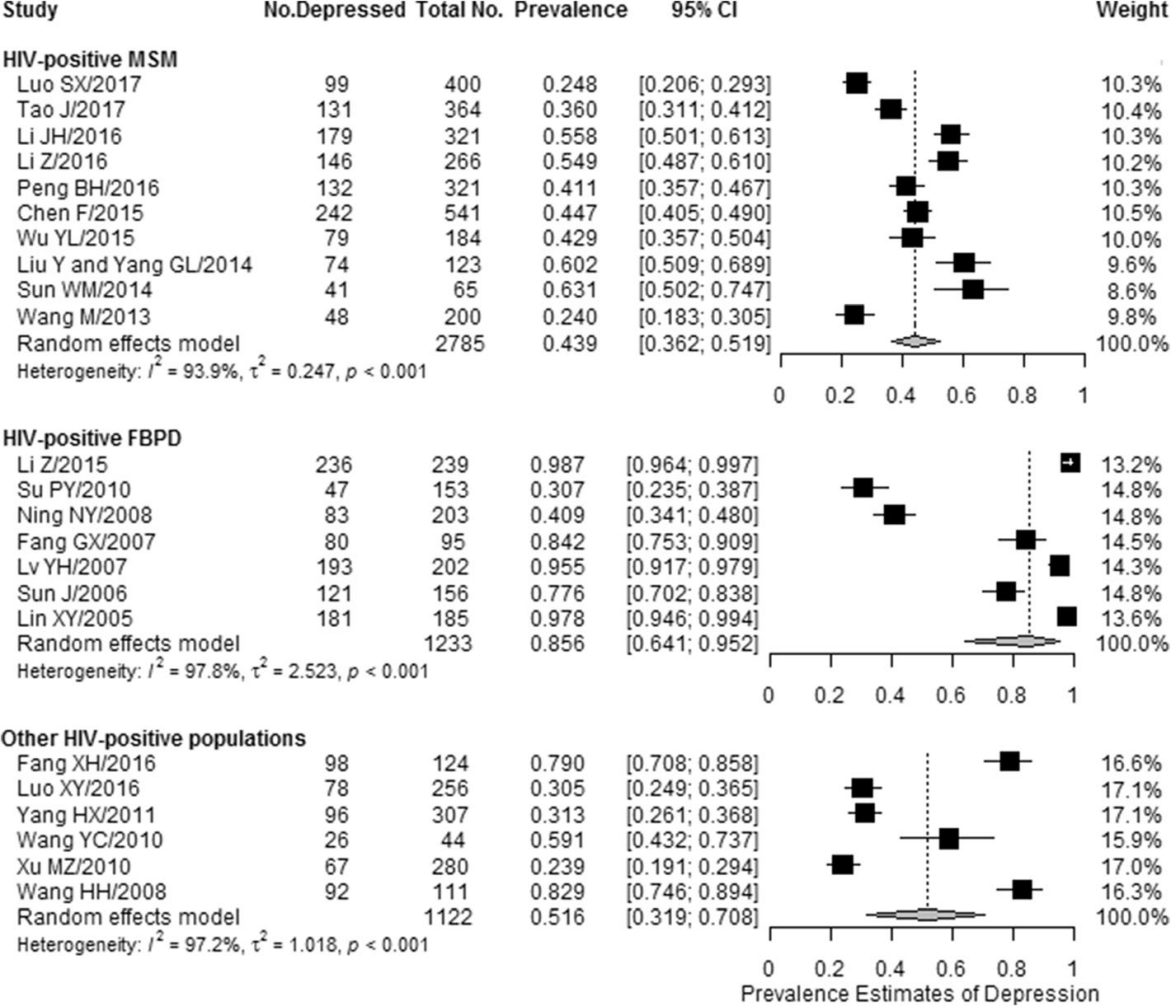
Forest plot of the prevalence of depression or depressive symptoms among the subgroups of people living with HIV/AIDS in China. The vertical dotted lines indicate the overall effect sizes of all studies combined within each sub-population who were living with HIV/AIDS in China. MSM, men who have sex with men; FBPD, former blood/plasma donors

The overall pooled prevalence of depression or depressive symptoms was 43.9% (1171/2785 individuals, 95% CI: 36.2–51.9%) among HIV-positive MSM, 85.6% (941/1233 individuals, 95% CI: 64.1–95.2%) among HIV-positive FBPD, and 51.6% (457/1122 individuals, 95% CI: 31.9–70.8%) among other HIV-positive populations. Significant heterogeneity was detected across studies in the prevalence estimates of depression or depressive symptoms in these specific sub-populations (*I*^*2*^ range: 93.9–97.8%; all *p <* 0.05) (Fig. 3).

## Discussion

In the present systematic review and meta-analysis, we quantified the proportion of depression or depressive symptoms among PLHA using data from seventy-four studies involving 20,635 individuals in seven areas of China. On average, the pooled prevalence estimates were 50.8% for depression or depressive symptoms among the general PLHA. We also quantified these proportion among specific PLHA. As significant heterogeneity was detected across studies for all these prevalence estimates, the results must be interpreted with caution. To the best of our knowledge, this study represents the first time that the epidemic of depression among PLHA in China was exhaustively reviewed. As depression among PLHA is a public health issue, the risk of burden on human resources and the health care systems is considerable. The study could help to estimate the public health burden of depression among PLHA in China and to guide policy, as well as advocacy efforts. Furthermore, the study represents the first step in developing effective interventions to prevent and treat associated sequelae.

Evidence suggested that the prevalence of depression among the general population in China ranged between 1.2 and 6.9% [103, 104], significantly lower than the prevalence rate reported in our study, which further confirmed that depression was an outcome conforming to logic among PLHA [105]. However, due to the common symptoms associated with HIV illness, such as pain, fatigue, insomnia, anorexia and cognitive impairment, it is difficult to diagnose depression among PLHA [106–110]. Based on a nationally representative sample, it is demonstrated that depression among PLHA is under-diagnosed in clinical practice in the United States [111]. Although there is no study on this issue in China, we can speculate that depression in the Chinese people with HIV/AIDS is also under-diagnosed in clinical practice because more than three-quarters of non-psychiatric clinicians in China lack adequate knowledge of depression [112], which has proven to contribute to the difficulty in identifying individuals with depression [113]. Moreover, a strong stigma against PLHA lead them to defer seeking health care services or to disclose their own HIV status to the health care workers [114], which is an additional obstacle to early detection and treatment of depression among PLHA. In fact, the serious shortness and uneven distribution of mental health resources are obstacles to directing adequate attention toward those health issues [115, 116]. To improve the current situation, the National Heath and Family Planning Commission of the People’s Republic of China issued the *Nation Mental Health Program (2015–2020)* [116] in 2015, in which a series of specific goals aimed at ultimately promoting public mental health have been proposed, including general improvement of the public cognition of depression and other common mental disorders and the public awareness of forwardly seeking medical advice, as well as obvious improvement in ability of medical workers to identify depression.

In our study, depression was found to be associated with the baseline survey time, on the decrease over time, even among some studies using common instruments. Economic development in the past decades may be a possible reason for this decrease in depression prevalence, which has greatly increased the investment of mental health as well as the availability of mental health services [115]. The growing awareness of AIDS-related knowledge among the public [117, 118] helps to reduce discrimination against PLHA and, hence, may be conductive to decreasing the prevalence of depression.

Even today, no consensus has been reached on the impact of ART on depression among PLHA in China. Several studies [54, 61, 68, 72, 78, 119, 120] have reported a higher prevalence of depression or depressive symptoms among PLHA who have undergone ART when compared with those who have not, and only two studies have reported a statistical significance [119, 120]. Nevertheless, some other studies [53, 58, 73, 121] have found the prevalence to be lower in patients who had received ART than in those who had not, while only one study has reported statistical significance for this opposite result [121]. Due to the lack of data available on depression prevalence estimates among the people using and not using ART, stratified meta-analyses could not be conducted in these two sub-populations. Instead, random-effects meta-regression analysis was used to explore the relationship between ART and depression or depressive symptoms prevalence. As a result, there was no significant association between them.

Given the higher reported prevalence estimates of depression among females in the general population, females were considered to be more vulnerable than males to the onset of depression [122], a finding supported by evidence from studies conducted in PLHA populations. In a observational cross-sectional study conducted in central India recruiting a large sample of 1181 PLHA, Deshmukh et al. have found that a greater percentage of females was screened positive for depressive symptoms when compared with males (59.9% vs 43.7%, *p* <  0.001) [123]. In another cross-sectional study conducted in Nigeria, a significantly higher prevalence of major depressive disorders was reported among females than among males [124]. However, in a current study targeted on newly diagnosed HIV-patients, being female was found to be protective against depression but without significance (OR = 0.48, *p* = 0.078) [125]. In our study, no significant association between the prevalence estimates of depression or depressive symptoms and gender was determined. In addition, in the general population, age has also been proven to be associated with variations in the prevalence estimates of depression, with younger participants having higher prevalence of current and lifetime depression than participants older than 50 or 55 years [126]. However, the association between age and depression among PLHA remained unclear. The results showed that a younger age was significant associated with the higher prevalence of depression screened by CES-D-20 [127], as well as diagnoses by psychiatrists, according to DSM-IV-TR [128], even after adjusting for confounding factors. However, the study conducted in HIV-infected adults undergoing anti-retroviral treatment demonstrated that participants older than 50 years old had a two times higher risk of depression when compared with participants with between 18 and 30 years old [129]. In addition, the result from the study which used the Depression, Anxiety, Stress subscales, and full Scale (DASS-21) for depression screening showed that no significant effect of age on the rate of depression was found among PHLA [123]. In this meta-analysis, although no significant association was found between age and the pooled prevalence of depression, age was demonstrated to be linked with a higher risk of depression in studies with Zung SDS scores ≥50 or CES-D-20 scores ≥16 as the criteria for screening positive, which might support the positive association between age and depression among PLHA to some extent. However, as there were few studies using those two screening instrument cut-offs as screening criteria, the results must be interpreted with caution. Further studies are needed to clarify the associations between gender/age and the risk of depression among PLHA, which will help to identify individuals in high-risk.

When interpreting the results of this study, note that the data synthesized in this meta-analysis were nearly entirely extract from studies using self-report inventories of depressive symptoms as the survey instruments, which had a wide range of sensitivity and specificity for diagnosing major depressive disorder (Additional file 4). Instruments such as the Psychological “Computerized Tomography” 4.0 Vision (PCT V4.0) have high specificity and sensitivity for diagnosing depression, whereas others instruments, such as the SCL-90, have low specificity and should be regarded as screening tools. Furthermore, evidences suggest that screening tools tend to over-estimate prevalence relative to diagnostic tools, which may lead to an over-estimation of true rates in the meta-analysis with all the included studies relying on screening instruments. Despite the limitations in self-report inventories of depressive symptoms, these inventories are still essential for assessing depression in HIV-positive individuals because they are easier and more cost-effective for use in busy specialty medical clinics and epidemiological surveys than formal diagnostic interviews [130, 131], particularly in epidemiological surveys. Because of the high prevalence in China, it is nearly impossible to assess depression through formal interviews between psychiatrists and HIV-positive individuals in epidemiological studies. As an alternative, self-report inventories are the best choice. Nevertheless, for primary care physicians, it is better to remember that the diagnosis of depression should not be based solely on the results of the screening questionnaire [132]. In this meta-analysis, to control the diversity in these inventories, stratified analyses were conducted based on survey instrument and cut-off scores that identified a range of prevalence estimates not presented in the previous review [133].

This study has important limitations. As with other meta-analyses, significant heterogeneity was found in the prevalence estimates in our study, which was incompletely explained by the stratified meta-analyses and meta-regressions analyses. We hypothesize that other variables might affect the heterogeneity, such as poor income adequacy, unemployment, homeless, lower CD4 counts, higher viral loads, the severity of depressive symptoms, duration of HIV/AIDS, poor self-efficacy and lack of social support. However, we were unable to obtain adequate information about these variables. For example, less than 1/3 of the studies reported the average or median counts of CD4 cells among HIV infected populations, and fewer than 10 studies provided employment-specific prevalence estimates of depression. In addition, although an extensive document retrieve was performed in multiple databases, the existence of non-indexed studies in those databases might have led to some relevant studies being ignored. Moreover, although an attempt was made to minimize the possible bias in the process of document retrieving with specific searches in major English-Chinese databases (including master and doctoral theses), there may still be some unidentified papers. Fortunately, as the results of Egger’s test results showed, there was no publication bias found in all results because we obtained a certain percentage of data from unpublished papers (fourteen theses [38, 56, 57, 66, 70, 71, 75, 82, 87, 89, 91, 94, 96, 99]).

## Conclusions

Our findings suggest that the estimates of depression or depressive symptoms among PLHA in China are considerable. Given that the progression of depression are associated with a higher short-term suicide risk and a higher long-term risk of cardiovascular disease and cancer [134, 135], the findings in this study highlight the need for screening and treatment for mental disorders to be integrated in the treatment package offered to PLHA, which will ultimately lead to better health outcomes for PLHA [136].

## Additional files

Additional file 1: “Search strategy used in the current systematic review and meta-analysis”. (DOC 66 kb)

Additional file 2: “Modified Newcastle-Ottawa risk of bias scoring guide”. (DOC 27 kb)

Additional file 3: “Selected characteristics of the 74 studies on the prevalence of depression or depressive symptoms among people living with HIV/AIDS in China”. (DOC 146 kb)

Additional file 4: “Modified Newcastle-Ottawa risk of bias score for the 74 studies included in this systematic review and meta-analysis”. (DOC 190 kb)

Additional file 5: “Sensitivities and specificities of commonly used instruments for diagnosing depression”. (DOC 43 kb)

Additional file 6: “Sensitivity analysis of the prevalence of depression or depressive symptoms among people living with HIV/AIDS in China”. (PDF 157 kb)

Additional file 7: “Within-instrument heterogeneity analyses of studies reporting on the prevalence of depression or depressive symptoms among people living with HIV/AIDS in China: stratified meta-analyses and meta-regression analyses”. (DOC 200 kb)

## Abbreviations

AIDS: acquired immune deficiency syndrome
ART: antiretroviral therapy
BDI: Beck Depression Inventory
CDC: Center for Disease Control and Prevention
CES-D-20: 20-item Center for Epidemiological Studies Depression Scale
CIs: confidence intervals
FBPD: former blood/plasma donors
HIV: human immunodeficiency virus
IDUs: injected drug users
MSM: men who have sex with men
NHIRD: National Health Insurance Research Database
NOS: Newcastle-Ottawa scale
PCT V4.0: Psychological “Computerized Tomography” 4.0 Vision
PLHA: people living with HIV/AIDS
SCL-90: 90-item Symptom Checklist
TB: tuberculosis
Zung SDS: Zung Self-Rating Depression Scale

## References

[1] World Health Organization. 10 facts on HIV/AIDS. 2017. http://www.who.int/features/factfiles/hiv/en/. Accessed 16 Sept 2017

[2] Chinese Center for Disease Control and Prevention (2015). Update on the AIDS/STD epidemic in China and main response in control and prevention in December, 2014. Chinese Journal of AIDS & STD..

[3] Chinese Center for Disease Control and Prevention (2017). Update on the AIDS/STD epidemic in China in the second quarter of 2017. Chinese Journal of AIDS & STD..

[4] Chinese Center for Disease Control and Prevention (2016). Update on the AIDS/STD epidemic in China and main response in control and prevention in December, 2015. Chinese Journal of AIDS & STD..

[5] Phillips KD, Sowell RL, Rojas M, Tavakoli A, Fulk LJ, Hand GA (2004). Physiological and psychological correlates of fatigue in HIV disease. Biol Res Nurs.

[6] Schuster R, Bornovalova M, Hunt E (2012). The influence of depression on the progression of HIV: direct and indirect effects. Behav Modif.

[7] Ciesla JA, Roberts J (2001). Meta-analysis of the relationship between HIV infection and risk for depressive disorders. Am J Psychiatry.

[8] Bing EG, Burnam MA, Longshore D, Fleishman JA, Sherbourne CD, London AS (2001). Psychiatric disorders and drug use among human immunodeficiency virus-infected adults in the United States. Arch Gen Psychiatry.

[9] Chen F, Li F, Chen L, Li Y, Li Y, Guo Z (2014). Analysis of depression and anxiety in HIV / AIDS patients. Journal of dermatology and. Venereology.

[10] Mayston R, Kinyanda E, Chishinga N, Prince M, Patel V (2012). Mental disorder and the outcome of HIV/AIDS in low-income and middle-income countries: a systematic review. AIDS.

[11] Carrico AW, Bangsberg DR, Weiser SD, Chartier M, Dilworth SE, Riley ED (2011). Psychiatric correlates of HAART utilization and viral load among HIV-positive impoverished persons. AIDS.

[12] Sumari-de Boer IM, Sprangers MA, Prins JM, Nieuwkerk PT (2012). HIV stigma and depressive symptoms are related to adherence and virological response to antiretroviral treatment among immigrant and indigenous HIV infected patients. AIDS Behav.

[13] Sabin CA, Ryom L, De Wit S, Mocroft A, Phillips AN, Worm SW (2013). Associations between immune depression and cardiovascular events in HIV infection. AIDS.

[14] Katon W, Schulberg H (1992). Epidemiology of depression in primary care. Gen Hosp Psychiatry.

[15] Bouhnik AD, Préau M, Vincent E, Carrieri MP, Gallais H, Lepeu G (2005). Depression and clinical progression in HIV-infected drug users treated with highly active antiretroviral therapy. Antivir Ther.

[16] Ammassari A, Antinori A, Aloisi MS, Trotta MP, Murri R, Bartoli L (2004). Depressive symptoms, neurocognitive impairment, and adherence to highly active antiretroviral therapy among HIV-infected persons. Psychosomatics.

[17] Kleeberger CA, Buechner J, Palella F, Detels R, Riddler S, Godfrey R (2004). Changes in adherence to highly active antiretroviral therapy medications in the multicenter AIDS cohort study. AIDS.

[18] Alciati A, Gallo L, Monforte AD, Brambilla F, Mellado C (2007). Major depression-related immunological changes and combination antiretroviral therapy in HIV-seropositive patients. Hum Psychopharmacol.

[19] Leserman J (2003). HIV disease progression: depression, stress, and possible mechanisms. Biol Psychiatry.

[20] Leserman J (2008). Role of depression, stress, and trauma in HIV disease progression. Psychosom Med.

[21] Burack JH, Barrett DC, Stall RD, Chesney MA, Ekstrand ML, Coates TJ (1993). Depressive symptoms and CD4 lymphocyte decline among HIV-infected men. JAMA.

[22] Leserman J, Petitto JM, Perkins DO, Folds JD, Golden RN, Evans DL (1997). Severe stress, depressive symptoms, and changes in lymphocyte subsets in human immunodeficiency virus-infected men. A 2-year follow-up study. Arch Gen Psychiatry.

[23] Vedhara K, Nott KH, Bradbeer CS, Davidson EA, Ong EL, Snow MH (1997). Greater emotional distress is associated with accelerated CD4+ cell decline in HIV infection. J Psychosom Res.

[24] Ironson G, O'Cleirigh C, Fletcher MA, Laurenceau JP, Balbin E, Klimas N (2005). Psychosocial factors predict CD4 and viral load change in men and women with human immunodeficiency virus in the era of highly active antiretroviral treatment. Psychosom Med.

[25] Patterson TL, Shaw WS, Semple SJ, Cherner M, McCutchan JA, Atkinson JH (1996). Relationship of psychosocial factors to HIV disease progression. Ann Behav Med.

[26] Page-Shafer K, Delorenze GN, Satariano WA, Winkelstein W Jr. Comorbidity and survival in HIV-infected men in the San Francisco Men's health survey. Ann Epidemiol 1996;6:420–430. 10.1016/s1047-2797(96)00064-6 8915473

[27] Ickovics JR, Hamburger ME, Vlahov D, Schoenbaum EE, Schuman P, Boland RJ (2001). Mortality, CD4 cell count decline, and depressive symptoms among HIV-seropositive women: longitudinal analysis from the HIV epidemiology research study. JAMA.

[28] Jiang Y, Wang M, Wei X, He J, Guo T, Huang G (2016). Prevalence of depression and related factors in 180 HIV/AIDS patients receiving highly active antiretroviral therapy. Zhonghua Liu Xing Bing Xue Za Zhi.

[29] Rong H, Nianhua X, Jun X, Lianguo R, Si W, Sheng W (2017). Prevalence of and risk factors for depressive symptoms among people living with HIV/AIDS receiving antiretroviral treatment in Wuhan, China: a short report. AIDS Care.

[30] Yao H, Zhu X, Wang H, Gu K (2014). Analysis of mental health status among people with HIV/AIDS in shanghai. Chinese Primary Health Care.

[31] Zhao L, Cai L, Cui W, Wang G, Zhao G, Prevalence HJ (2017). Influencing factors of depression and anxiety among people living with HIV/AIDS in Yunnan province. Chin J Public Health.

[32] Rotenstein LS, Ramos MA, Torre M, Segal JB, Peluso MJ, Guille C (2016). Prevalence of depression, depressive symptoms, and suicidal ideation among medical students: a systematic review and Meta-analysis. JAMA.

[33] Moher D, Liberati A, Tetzlaff J, Altman D, PRISMA Group. Preferred reporting items for systematic reviews and meta-analyses: the PRISMA statement. Open Med 2009;3:e123–e130. PMC3090117 21603045

[34] Tao J, Vermund S, Lu H, Ruan Y, Shepherd B, Kipp A (2017). Impact of depression and anxiety on initiation of antiretroviral therapy among men who have sex with men with newly diagnosed HIV infections in China. AIDS Patient Care STDs.

[35] Hu J, Zhou X, Qu F, Xu T, Wang S, Yi Y (2017). Depression of HIV/AIDS outpatients and its influence factors. Journal of Nursing.

[36] Huang X, Li H, Meyers K, Xia W, Meng Z, Li C (2017). Burden of sleep disturbances and associated risk factors: a cross-sectional survey among HIV-infected persons on antiretroviral therapy across China. Sci Rep.

[37] Luo S, Zhou H, Zhou C, Li Y (2017). The psychological health status and sexual behavior in the HIV-infected men who have sex with men. Journal of Preventive Medicine Information.

[38] Mo X. Investgate the psychological distress status and evaluate the effect of individualized psychological intervention in HIV/AIDS. Guangxi medical university (Nanning); 2017. MA thesis.

[39] Wang H, Wang M, Jiang F, Liu Z, Wang H, Li J (2017). Socio-psychological factors relevant to suicidal ideation among patients with AIDS in Changsha. Zhong Nan Da Xue Xue Bao Yi Xue Ban.

[40] Yu N (2017). The effects of beliefs about AIDS-related death on quality of life in Chinese married couples with both husband and wife infected with HIV: examining congruence using the actor-partner interdependence model. Health Qual Life Outcomes.

[41] Zhou J, Cheng H, Xu F, Zhang X, Zhu J, Wang P (2016). The psychological and social support status of people living with HIV/AIDS in Wuxi. Chinese Journal of Disease Control & Prevention.

[42] Fang X, Ma D, Zou Z, Kan X, Tang L (2016). Social support and mental health among patients with TB/HIV double infection in Anhui Province. Chinese Rural Health Service Administration.

[43] Li C, Chen Y, Xing J, Zhang Q, Xiong B, Zhao Z (2016). Investigation of psychological health of people living with HIV/AIDS in Dalian. Studies of Trace Elements and Health.

[44] Li J, Mo PKH, Kahler CW, Lau JTF, Du M, Dai Y (2016). Prevalence and associated factors of depressive and anxiety symptoms among HIV-infected men who have sex with men in China. AIDS Care.

[45] Li L, Lyu C, Zhang X, Dong L, Fu J (2016). Survey on quality of life among AIDS patients who received ART. Jinan city Preventive Medicine Tribune.

[46] Li Y, Song X, Zhao X, Li T (2016). The level of anxiety and depression in HIV/AIDS patients receiving highly active antiretroviral therapy. J Nurs Adm.

[47] Li Z, Hsieh E, Morano JP, Sheng Y (2016). Exploring HIV-related stigma among HIV-infected men who have sex with men in Beijing, China: a correlation study. AIDS Care.

[48] Luo X, Jimu M, Yang Q, Shama X (2016). Analysis of depressive status of AIDS patients co-infected with tuberculosis in Liangshan Yi autonomous prefecture. Chinese Journal of AIDS & STD..

[49] Peng B, Luo D, Liu Y, Niu L, Wang M, Chen X (2016). HIV/AIDS stress, emotional problems and social support among newly HIV-infected men who have sex with men. China Journal of Modern Medicine..

[50] Zhang H, Zeng C, Cai W, Guo Z, Zhu Y, Sun Y (2016). Suicidal status and related factors between male and female people living with HIV/AIDS. Chinese Journal of AIDS & STD..

[51] Zhang X, Tan W, Zhang Z, Wu D, Xu H, Sun Y (2016). A follow-up survey on mental health of people with HIV/AIDS in the early stage of antiviral therapy. Journal of Preventive Medicine of Chinese People's Liberation Army.

[52] Zhao C, Zhou Z (2016). Psychological status of HIV/AIDS patients and their relatives in Shufu county of Xinjiang. Chinese Journal of AIDS & STD..

[53] Chen F, Ding F, Lin X, Wang X, He H, Huang W (2015). Prevalence rates of depression and anxiety in HIV-infected men who have sex with men. Chin Ment Health J.

[54] Guo Z, Cai W, Zhou Q, Zhu Y, Guo Y. Gender differences in factors associated with depression among people living with HIV/AIDS in Guangzhou. South China. J Prev Med. 2015;41:501–6.

[55] Li X, Li L, Wang H, Fennie KP, Chen J, Williams AB (2015). Mediation analysis of health-related quality of life among people living with HIV infection in China. Nurs Health Sci.

[56] Li Z. Investigation on the status of perceived discrimination in HIV / AIDS patients and its influencing factors. Peking union medical college (Beijing); 2015. MA thesis.

[57] Sun HM. Adherence to antiretroviral therapy, anxiety and depression of AIDS patients in Anhui, China. Anhui Medical University (Hefei); 2015. MA thesis.

[58] Wu Y, Yang H, Wang J, Yao H, Zhao X, Chen J (2015). Prevalence of suicidal ideation and associated factors among HIV-positive MSM in Anhui, China. Int J STD AIDS.

[59] Wang H, Zhang C, Ruan Y, Li X, Fennie K, Williams A (2014). Depressive symptoms and social support among people living with HIV in Hunan, China. J Assoc Nurses AIDS Care.

[60] Qiu Y, Luo D, Cheng R, Xiao Y, Chen X, Huang Z (2014). Emotional problems and related factors in patients with HIV/AIDS. Zhong Nan Da Xue Xue Bao Yi Xue Ban.

[61] Dwyer R, Wenhui L, Cysique L, Brew BJ, Lal L, Bain P (2014). Symptoms of depression and rates of neurocognitive impairment in HIV positive patients in Beijing, China. J Affect Disord.

[62] Hou WL, Chen CE, Liu HY, Lai YY, Lee HC, Lee NY (2014). Mediating effects of social support on depression and quality of life among patients with HIV infection in Taiwan. AIDS Care.

[63] Liu H, He X, Levy JA, Xu Y, Zang C, Lin X (2014). Psychological impacts among older and younger people living with HIV/AIDS in Nanning, China. J Aging Res.

[64] Liu Y (2014). Impact of perceived stigma of HIV/AIDS patients on mental health and unsafe sexual behavior. China Journal of Modern Medicine.

[65] Liu Y, Yang G, Gong H, Yan J (2014). Association of perceived stigma, psychological status and sexual behavior in the HIV-infected persons among men who have sex with men. Chinese Journal of Modern Medicine.

[66] Peng M. Research on current situation of medical discrimination against person living with HIV/AIDS (PLWHA) in Guangdong province. Jinan University (Guangzhou); 2014. MA thesis.

[67] Shi K, He Y, Zou Y (2014). Personal and family's factors influencing the self-discrimination of AIDS patients. Chinese Journal of Disease Control & Prevention..

[68] Sun W, Wu M, Qu P, Lu C, Wang L (2014). Psychological well-being of people living with HIV/AIDS under the new epidemic characteristics in China and the risk factors: a population-based study. Int J Infect Dis.

[69] Sun W, Lu L, Yuan Y, Xu D, Chen X, Zou Q (2014). The anxiety and depression of male homosexual with HIV/AIDS in Nanchang City: current situation and related factors. Chinese Journal of Disease Control & Prevention..

[70] Yang G. Retention in care and its related factors of people living with HIV/AIDS in Changsha. Central South University (Changsha); 2014. MA thesis.

[71] Zhou G. A survey on psychological states and coping styles in HIV/AIDS during the antiretroviral therapy in three hospitals in Yunnan Province. Kunming Medical University (Kunming); 2014. MA thesis.

[72] Zhou Z, Gao Y (2014). Mental health of AIDS/HIV positive clients. China Journal of Health Psychology.

[73] Liu L, Pang R, Sun W, Wu M, Qu P, Lu C (2013). Functional social support, psychological capital, and depressive and anxiety symptoms among people living with HIV/AIDS employed full-time. BMC Psychiatry.

[74] Su X, Lau JTF, Mak WWS, Choi KC, Chen L, Song J (2013). Prevalence and associated factors of depression among people living with HIV in two cities in China. J Affect Disord.

[75] Wang M. Health status and health service utilization in a sample of HIV-positive men who have sex with men in shanghai. Anhui Medical University (Hefei); 2013. MA thesis.

[76] Yang Y, Qin X, Zhang H, Huang G, Li G, Li Y (2013). The study on depression and influencing factors among people living with HIV/AIDS. Journal of Tropical Medicine.

[77] Bo P (2012). The evaluation of degree of depression and quality of life of HIV/AIDS patients. Chinese and Foreign Medical Research.

[78] Rao D, Chen WT, Pearson CR, Simoni JM, Fredriksen-Goldsen K, Nelson K (2012). Social support mediates the relationship between HIV stigma and depression/quality of life among people living with HIV in Beijing, China. Int J STD AIDS.

[79] Yeh ML, Hsu ST, Ko WC, Ko NY (2012). Depressive symptoms in people living with HIV: related factors. Hu li za zhi.

[80] Dong W, Geng W, Huang J, Qin Y, Ou R, Qin Y (2011). Multi-variate logistic regression analysis of influencing factors of the depression in the HIV-infected out-patients. Chinese journal of new Clinical Medicine.

[81] Sun Y, Fan A, Yan X (2011). Survey on psychology status and related factors among population with HIV/ AIDS. Chinese journal of public. Health Management.

[82] Yang HX. Research on the stress and its correlates of HIV-infected pregnant women. Kunming Medical University (Kunming); 2011. MA thesis.

[83] Liu T, Fu J, Kang D, Qian Y, Lyu C, Zhang X (2010). Investigation on living condition among HIV-infected patients in Shandong Province. Preventive Medicine Tribune.

[84] Lu L, He C, Yang Y (2010). Analysis and persuasion of depression during AIDS patients in rural areas. China Modern Doctor.

[85] Su P, Tao F, Hao J, Huang K, Zhu P (2010). Mental health and risk behavior of married adult HIV/AIDS subjects derived from paid blood donation in the rural of Anhui Province. Journal of Hygiene Research.

[86] Wang Y, Li AA (2010). Study on depression, anxiety and related factors in pregnant women infected with human immunodeficiency virus. Chinese Journal of Family Planning.

[87] Xu MZ. Attempted suicide and mental disorders among HIV-positive drug users in Guangdong. Guangdong pharmaceutical university (Guangzhou); 2010. MA thesis.

[88] Jin C, Zhao G, Zhang F, Feng L (2009). Wu N. The psychological status of HIV-positive people and their psychosocial experiences in eastern China. HIV MED.

[89] Chen G. Study of the mental health and the influencing factors with rural area PLWHA in a county of Henan. Third Military Medical University (Chongqing); 2008. MA thesis.

[90] Li B, Zhu Y, Mo J, Zhang J (2008). Psychological evaluation of anxiety and depression response for 95 cases of HIV positive people. Journal of Kunming Medical University.

[91] Li J. Study on PLWHA demand for psychological care evaluation and intervention in rural area. Anhui Medical University (Hefei); 2008. MA thesis.

[92] Ning N, Shi C, Yu X, Chen Y, Wu Z, Jin H (2008). Depression and quality of life investigation among HIV/AIDS subjects infected by plasma donation. Chin Ment Health J.

[93] Wang H, Zhou J, Huang L (2008). Adherence to highly active antiretroviral treatment and related factors in drug users with HIV/AIDS. Chinese journal of behavioral medicine and brain. Science.

[94] Zhu XY. Research and evaluation on the psychology and quality of life among HIV/AIDS cases and correlated people. Shandong Academy of Medical Sciences (Jinan); 2008. MA thesis.

[95] Fang G, Jiang Q, Dong Y (2007). Analysis and study of psychological state among paid blood donors infected with HIV in rural areas. Chinese Journal of Disease Control & Prevention..

[96] Lyu YH. A cross-sectional study on the mental health among people living with HIV/AIDS. Peking union medical college (Beijing); 2007. MA thesis.

[97] Huang T, Leu H, Liu J (2006). Lymphocyte subsets and viral load in male AIDS patients with major depression: naturalistic study. Psychiatry Clin Neurosci.

[98] Jin H, Hampton Atkinson J, Yu X, Heaton RK, Shi C, Marcotte TP (2006). Depression and suicidality in HIV/AIDS in China. J Affect Disord.

[99] Sun J. Study examines the living situation of people living with HIV/AIDS in Suizhou City of Hubei Province. Huazhong University of Science and Technology (Wuhan); 2006. MA thesis.

[100] Lin X, Wu H, Zhang T, Fu Y, Wang J (2005). Analysis of depression and anxiety among paid blood donors infected with HIV in rural areas. Chinese Journal of AIDS & STD..

[101] Liao J, Ma Y, Xiong J, Kuang W, Shi Q, Xiao Y (2004). Investigation of depressive status of patients with HIV/AIDS and their family members. Chinese. J Dermatol.

[102] Yen CF, Tsai JJ, Lu PL, Chen YH, Chen TC, Chen PP (2004). Quality of life and its correlates in HIV/AIDS male outpatients receiving highly active antiretroviral therapy in Taiwan. Psychiatry Clin Neurosci.

[103] Luo H, Li Y (2012). Study on the status quo of the domestic depression disorder prevalence and its distribution characteristics. Foreign Medical Sciences (Section of Medgeography).

[104] Lu J, Ruan Y, Huang Y, Yao J, Dang W, Gao C (2008). Major depression in Kunming: prevalence, correlates and co-morbidity in a south-western city of China. J Affect Disord.

[105] Bernard C, Dabis F, Prevalence d RN (2017). Factors associated with depression in people living with HIV in sub-Saharan Africa: a systematic review and meta-analysis. PLoS One.

[106] Benton TD (2008). Depression and HIV/AIDS. Curr Psychiatry Rep.

[107] Kalichman SC, Rompa D, Cage M (2000). Distinguishing between overlapping somatic symptoms of depression and HIV disease in people living with HIV-AIDS. J Nerv Ment Dis.

[108] Gibbie T, Mijch A, Ellen S, Hoy J, Hutchison C, Wright E (2006). Depression and neurocognitive performance in individuals with HIV/AIDS: 2-year follow-up. HIV Med.

[109] Castellon SA, Hardy DJ, Hinkin CH, Satz P, Stenquist PK, van Gorp WG (2006). Components of depression in HIV-1 infection: their differential relationship to neurocognitive performance. J Clin Exp Neuropsychol.

[110] Grant I (2008). Neurocognitive disturbances in HIV. Int Rev Psychiatry.

[111] Asch SM, Kilbourne AM, Gifford AL, Burnam MA, Turner B, Shapiro MF (2003). Underdiagnosis of depression in HIV: who are we missing. J Gen Intern Med.

[112] Liang H, Fei L, Zhang Y, Xue D, Wei L, Cui Y (2007). A preliminary investigation on the knowledge of depression in non-psychiatric clinic staff in three cities in developing countries. Chinese Journal of Nervous and Mental Diseases.

[113] Akena D, Stein DJ, Joska J (2013). Does screening HIV-positive individuals in Uganda for major depressive disorder improve case detection rates and antidepressant prescription?. AIDS Behav.

[114] Zhang X, Miège P, Zhang Y (2011). Decentralization of the provision of health services to people living with HIV/AIDS in rural China: the case of three counties. Health Res Policy Syst.

[115] Jiang Z (2016). Research progress on the status quo and needs of mental health resources in China. Journal of Youjiang Medical University for Nationalities.

[116] National Heath and Family Planning Commission of the People's Republic of China. National mental health program (2015 - 2020). 2015. http://www.gov.cn/zhengce/content/2015-06/18/content_9860.htm. Accessed 16 Sept 2017

[117] Du W, Guo L, Wei N, Tao M, Tian X, An J (2011). An analysis of AIDS related knowledge among Chinese citizens and its influencing factors. Chinese Journal of AIDS & STD..

[118] Zhang X, Su X, Song W, Wang C, Wang L, Zhang L (2013). Telephone survey on awareness of HIV/AIDS and related policy among residents in 19 provinces of China. Chinese Journal of AIDS & STD.

[119] Liu Q, Yang JJ, Guo Y (2011). Investigation on depression and social support among people living with HIV/AIDS. Medical Journal of Wuhan University.

[120] Li L. A study on living status of people living with HIV/AIDS in high HIV prevalence districts. Peking union medical college (Beijing); 2007. MA thesis.

[121] Wang H, Zhou J, Huang L, Li X (2008). Adherence to highly active antiretroviral therapy and quality of life in patients with acquired immunodeficiency syndrome. Chinese Journal of Nursing.

[122] Richards D (2011). Prevalence and clinical course of depression: a review. Clin Psychol Rev..

[123] Deshmukh NN, Borkar AM, Deshmukh JS (2017). Depression and its associated factors among people living with HIV/AIDS: can it affect their quality of life?. J Family Med Prim Care.

[124] Egbe CO, Dakum PS, Ekong E, Kohrt BA, Minto JG, Ticao CJ (2017). Depression, suicidality, and alcohol use disorder among people living with HIV/AIDS in Nigeria. BMC Public Health.

[125] L'Akoa RM, Noubiap JJ, Fang Y, Ntone FE, Kuaban C (2013). Prevalence and correlates of depressive symptoms in HIV-positive patients: a cross-sectional study among newly diagnosed patients in Yaoundé, Cameroon. BMC Psychiatry..

[126] Kessler RC, Berglund P, O D, Merikangas KR, Rush AJ, Walters EE, et al. The epidemiology of major depressive disorder: results from the National Comorbidity Survey Replication (NCS-R). JAMA 2003;289:3095–3105. 10.1001/jama.289.23.3095 12813115

[127] Seth P, Kidder D, Pals S, Parent J, Mbatia R, Chesang K (2014). Psychosocial functioning and depressive symptoms among HIV-positive persons receiving care and treatment in Kenya, Namibia, and Tanzania. Prev Sci.

[128] Akena D, Musisi S, Joska J, Stein DJ (2012). The association between aids related stigma and major depressive disorder among HIV-positive individuals in Uganda. PLoS One.

[129] Kaharuza FM, Bunnell R, Moss S, Purcell DW, Bikaako-Kajura W, Wamai N (2006). Depression and CD4 cell count among persons with HIV infection in Uganda. AIDS Behav.

[130] Vilagut G, Forero CG, Barbaglia G, Alonso J (2016). Screening for depression in the general population with the Center for Epidemiologic Studies Depression (CES-D): a systematic review with Meta-analysis. PLoS One.

[131] Wang YP, Gorenstein C (2013). Assessment of depression in medical patients: a systematic review of the utility of the Beck depression inventory-II. Clinics (Sao Paulo).

[132] Kerr LK, Kerr LD Jr. Screening tools for depression in primary care: the effects of culture, gender, and somatic symptoms on the detection of depression. West J Med 2001;175:349–352. 10.1136/ewjm.175.5.349 PMC1071624 11694495

[133] Niu L, Luo D, Liu Y, Silenzio VM, Xiao S (2016). The mental health of people living with HIV in China, 1998-2014: a systematic review. PLoS One.

[134] Clarke DM, Depression CK (2009). Anxiety and their relationship with chronic diseases: a review of the epidemiology. risk and treatment evidence Med J Aust.

[135] Glassman AH, Shapiro PA (1998). Depression and The course of coronary artery disease. Am J Psychiatry.

[136] Gaynes BN, O'Donnell J, Nelson E, Heine A, Zinski A, Edwards M (2015). Psychiatric comorbidity in depressed HIV-infected individuals: common and clinically consequential. Gen Hosp Psychiatry.

