# Supplementary material for: Prevalence of depression or depressive symptoms among people living with HIV/AIDS in China: a systematic review and meta-analysis
Source: BMC Psychiatry. 2018 May 31;18:160. doi: 10.1186/s12888-018-1741-8 (PMC5984474; doi:10.1186/s12888-018-1741-8)
Supplement: Supplementary file 1 — “Search strategy used in the current systematic review and meta-analysis”. (DOC 66 kb) [file 12888_2018_1741_MOESM1_ESM.doc]

Additional file 1 Search strategy used in the current systematic review and meta-analysis

| Source | Search strategy |
| --- | --- |
| 1. PubMed | 1-human immunodeficiency virus[Title/Abstract] |
|  | 2-acquired immune deficiency syndrome[Title/Abstract] |
|  | 3-HIV[Title/Abstract] |
|  | 4-AIDS[Title/Abstract] |
|  | 5-#1 OR #2 OR #3 OR #4 |
|  | 6-depression[Title/Abstract] |
|  | 7-depressive disorder[Title/Abstract] |
|  | 8-depressive symptom[Title/Abstract] |
|  | 9-mental disorder[Title/Abstract] |
|  | 10-mental health[Title/Abstract] |
|  | 11-mood disorder[Title/Abstract] |
|  | 12-affective disorder[Title/Abstract] |
|  | 13-psychological health[Title/Abstract] |
|  | 14-psychiatric[Title/Abstract] |
|  | 15-#6 OR #7 OR #8 OR #9 OR #10 OR #11 OR #12 OR #13 OR #14 |
|  | 16-#5 AND #15 |
| 1. Embase |  |
|  | 1-'human immunodeficiency virus':ab,ti |
|  | 2-'acquired immune deficiency syndrome':ab,ti |
|  | 3-'hiv':ab,ti |
|  | 4-'aids':ab,ti |
|  | 5-#1 OR #2 OR #3 OR #4 |
|  | 6-'depression':ab,ti |
|  | 7-'depressive disorder':ab,ti |
|  | 8-'depressive symptom':ab,ti |
|  | 9-'mental disorder':ab,ti |
|  | 10-'mental health':ab,ti |
|  | 11-'mood disorder':ab,ti |
|  | 12-'affective disorder':ab,ti |
|  | 13-'psychological health':ab,ti |
|  | 14-'psychiatric':ab,ti |
|  | 15-#6 OR #7 OR #8 OR #9 OR #10 OR #11 OR #12 OR #13 OR #14 |
|  | 16-#5 AND #15 |
| 1. Web of Science (All Databases) |  |
|  | 1-TOPIC: (human immunodeficiency virus) |
|  | 2-TOPIC: (acquired immune deficiency syndrome) |
|  | 3-TOPIC: (HIV) |
|  | 4-TOPIC: (AIDS) |
|  | 5-#1 OR #2 OR #3 OR #4 |
|  | 6-TOPIC: (depression) |
|  | 7-TOPIC: (depressive disorder) |
|  | 8-TOPIC: (depressive symptom) |
|  | 9-TOPIC: (mental disorder) |
|  | 10-TOPIC: (mental health) |
|  | 11-TOPIC: (mood disorder) |
|  | 12-TOPIC: (affective disorder) |
|  | 13-TOPIC: (psychological health) |
|  | 14-TOPIC: (psychiatric) |
|  | 15-#6 OR #7 OR #8 OR #9 OR #10 OR #11 OR #12 OR #13 OR #14 |
|  | 16-#5 AND #15 |
| 1. China National Knowledge Infrastructure |  |
|  | 1-人类免疫缺陷病毒（主题）或 获得性免疫缺陷综合征（主题）或 艾滋病（主题）或 HIV（主题）或 AIDS（主题） |
|  | 2-结果中检索：抑郁（主题）或 精神障碍（主题）或 精神卫生（主题）或 情绪失调（主题）或 情感障碍（主题）或 心理卫生（主题） |
| 1. Wanfang Database |  |
|  | (主题:(∷人类免疫缺陷病毒∷)+主题:(∷获得性免疫缺陷综合征∷)+主题:(∷艾滋病∷)+主题:(∷HIV∷)+主题:(∷AIDS∷))*((∷抑郁∷)+主题:(∷精神障碍∷)+主题:(∷精神卫生∷)+主题:(∷情绪失调∷)+主题:(∷情感障碍∷)+主题:(∷心理卫生∷)) |
| 1. Weipu | 1-文摘=人类免疫缺陷病毒 |
|  | 2-文摘=获得性免疫缺陷综合征 |
|  | 3-文摘=艾滋病 |
|  | 4-文摘=HIV |
|  | 5-文摘=AIDS |
|  | 6-#1 OR #2 OR #3 OR #4 OR #5 |
|  | 7-文摘=抑郁 |
|  | 8-文摘=精神障碍 |
|  | 9-文摘=精神卫生 |
|  | 10-文摘=情绪失调 |
|  | 11-文摘=情感障碍 |
|  | 12-文摘=心理卫生 |
|  | 13-#7 OR #8 OR #9 OR #10 OR #11 OR #12 |
| 1. China Biology Medicine disc |  |
|  | ((((("人类免疫缺陷病毒"[常用字段:智能]) OR "获得性免疫缺陷综合征"[常用字段:智能]) OR "艾滋病"[常用字段:智能]) OR "HIV"[常用字段:智能]) OR "AIDS"[常用字段:智能]) AND (((((("抑郁"[常用字段:智能]) OR "精神障碍"[常用字段:智能]) OR "精神卫生"[常用字段:智能]) OR "情绪失调"[常用字段:智能]) OR "情感障碍"[常用字段:智能]) OR "心理卫生"[常用字段:智能]) |
