# Supplementary material for: Prevalence of depression or depressive symptoms among people living with HIV/AIDS in China: a systematic review and meta-analysis
Source: BMC Psychiatry. 2018 May 31;18:160. doi: 10.1186/s12888-018-1741-8 (PMC5984474; doi:10.1186/s12888-018-1741-8)
Supplement: Supplementary file 2 — “Modified Newcastle-Ottawa risk of bias scoring guide”. (DOC 27 kb) [file 12888_2018_1741_MOESM2_ESM.doc]

**Additional file 2** Modified Newcastle-Ottawa risk of bias scoring guide.

1. **Sample representativeness:**

1 point: the study population contained HIV-positive individuals who had been recruited from the registered HIV-infected individuals databases of the provincial or municipal Center for Disease Control and Prevention (CDC), or from multiple study sites.

0 point: subjects were HIV-positive individuals who had recruited only from methadone clinic or the HIV clinic.

1. **Sample size:**

1 point: sample size was greater than or equal to 100 individuals.

0 point: sample size was less than 100 individuals.

1. **Response rate:**

1 point: a response rate was greater than or equal to 70% was reported.

0 point: there was no response rate reported or a response rate less than 70% reported.

1. **Ascertainment of depression:**

1 point: depression has been detected by clinical interview or a commonly used measurement tool with a valid cutoff score (e.g., Zung SDS ≥ 50, HADS-D ≥ 8, PHQ-9 ≥ 10, CESD-20 ≥ 20, BDI ≥ 10).

0 point: depression has been detected by an infrequently used measurement tool, a commonly measurement tool with an invalid cutoff score, or any tool with published specificity/sensitivity values less than 70% (e.g., BDI ≥ 5, Zung SDS ≥ 53).

1. **Quality of descriptive statistics reporting:**

1 point: the study reported descriptive statistics to describe people living with HIV (e.g., age, sex) with proper measures of dispersion (e.g., mean ± standard deviation, median & 25^th^ to 75^th^ percentile ).

0 point: the study did not report or incompletely reported descriptive statistics, or reported without proper measures of dispersion.

**NEWCASTLE - OTTAWA QUALITY ASSESSMENT SCALE**

**COHORT STUDIES**

Note: A study can be awarded a maximum of one star for each numbered item within the Selection and Outcome categories. A maximum of two stars can be given for Comparability

**Selection**

1) Representativeness of the exposed cohort

a) truly representative of the average _______________ (describe) in the community ****

b) somewhat representative of the average ______________ in the community ****

c) selected group of users eg nurses, volunteers

d) no description of the derivation of the cohort

2) Selection of the non exposed cohort

a) drawn from the same community as the exposed cohort ****

b) drawn from a different source

c) no description of the derivation of the non exposed cohort

3) Ascertainment of exposure

a) secure record (eg surgical records) ****

b) structured interview ****

c) written self report

d) no description

4) Demonstration that outcome of interest was not present at start of study

a) yes ****

b) no

**Comparability**

1) Comparability of cohorts on the basis of the design or analysis

a) study controls for _____________ (select the most important factor) ****

b) study controls for any additional factor **** (This criteria could be modified to indicate specific control for a second important factor.)

**Outcome**

1) Assessment of outcome

a) independent blind assessment ****

b) record linkage ****

c) self report

d) no description

2) Was follow-up long enough for outcomes to occur

a) yes (select an adequate follow up period for outcome of interest) ****

b) no

3) Adequacy of follow up of cohorts

a) complete follow up - all subjects accounted for ****

b) subjects lost to follow up unlikely to introduce bias - small number lost - > ____ % (select an adequate %) follow up, or description provided of those lost) ****

c) follow up rate < ____% (select an adequate %) and no description of those lost

d) no statement
