# Supplementary material for: Prevalence of depression or depressive symptoms among people living with HIV/AIDS in China: a systematic review and meta-analysis
Source: BMC Psychiatry. 2018 May 31;18:160. doi: 10.1186/s12888-018-1741-8 (PMC5984474; doi:10.1186/s12888-018-1741-8)
Supplement: Supplementary file 3 — “Selected characteristics of the 74 studies on the prevalence of depression or depressive symptoms among people living with HIV/AIDS in China”. (DOC 146 kb) [file 12888_2018_1741_MOESM3_ESM.doc]

**Additional file 3** Selected characteristics of the 74 studies on the prevalence of depression or depressive symptoms among people living with HIV/AIDS in China

| Study | Survey time | Geographic location | Subjects | Sample size | Age, y | Men, no. (%) | Instrument and cutoff score |
| --- | --- | --- | --- | --- | --- | --- | --- |
| Hu J/2017 | 2015.07-2015.10 | Hunan | HIV-positive men and women | 360 | Mean (SD): 35.6 (11.5) | 285 (79.2) | Zung SDS ≥ 50 |
| Huang XJ/2017 | 2014.01-2015.12 | Beijing, Guangxi, Yunnan and other major municipalities | HIV-positive men and women | 4,103 | Mean (SD): 37.6 (11.7) | 3204 (78.1) | HADS-D ≥ 8 |
| Mo XY/2017 | 2015.10-2016.05 | Guangxi | HIV-positive men and women | 321 | Mean (SD): 46.6 (13.7) | 218 (67.9) | Zung SDS ≥ 53 |
| Rong H/2017 | 2015.10-2015.12 | Hubei | HIV-positive men and women | 394 | NR | 363 (92.1) | BDI-II ≥ 14 |
| Wang HY/2017 | 2015.12-2016.01 | Hunan | HIV-positive men and women | 504 | Mean (SD): 35.3 (11.6) | 409 (91.2) | Zung SDS SI ≥ 0.5 |
| Zhao L/2017 | 2016.01-2016.02 | Yunnan | HIV-positive men and women | 410 | Mean (SD): 41.7 (11.5) | 291 (71.0) | PHQ-9 ≥ 5 |
| Jiang Y/2016 | 2015.06-2015.12 | Hunan | HIV-positive men and women | 180 | Mean (SD): 37.2 (10.0) | 131 (72.8) | PCT V4.0 |
| Li C/2016 | 2012.06 | Liaoning | HIV-positive men and women | 150 | Mean (SD): 36.6 (12.6) | 102 (68.0) | SCL-90 ≥ 2 |
| Li L/2016 | 2016.03-2016.07 | Shandong | HIV-positive men and women | 166 | Mean (SD): 31.4 (9.4) | 164 (98.8) | Zung SDS ≥ 53 |
| Li YL/2016 | 2014.01-2014.06 | Beijing | HIV-positive men and women | 118 | Median (range): 35.3 (15-91) | 82 (69.5) | HADS-D ≥ 8 |
| Sun YP/2016 | 2013.03-2013.06 | Guangdong | HIV-positive men and women | 406 | Mean (SD): 35.5 (8.8) | 284 (70) | CES-D-20 ≥ 20 |
| Zhang CH/2016 | 2015 | Xinjiang | HIV-positive men and women | 103 | Mean (range): 26 (24-37) | 50 (48.5) | SCL-90 ≥ 2 |
| Zhang HX/2016 | 2013.03-2013.06 | Guangzhou | HIV-positive men and women | 408 | NR | 284 (69.6) | CES-D-20 ≥ 20 |
| Zhang X/2016 | 2011.04-2013.03 | Beijing | HIV-positive men and women | 57 | Mean (SD): 38.6 (10.9) | 52 (91.2) | SCL-90 ≥ 2 |
| Zhou J/2016 | 2015.08-2015.10 | Jiangsu | HIV-positive men and women | 110 | Median: 34.4 | 105 (95.5) | SCL-90 ≥ 2 |
| Guo ZH/2015 | 2013.03-2013.05 | Guangdong | HIV-positive men and women | 409 | Mean (SD): 36.3 (8.8) | 286 (69.9) | CES-D-20 ≥ 17 |
| Li XH/2015 | 2010.07-2011.08 | Hunan | HIV-positive men and women | 114 | Mean (SD): 39 (10.2) | 81 (71.1) | CES-D-20 ≥ 16 |
| Sun HM/2015 | 2013.01-2014.06 | Anhui | HIV-positive men and women | 298 | Mean (SD): 42.4 (10.6) | 199 (66.8) | Zung SDS ≥ 53 |
| Chen FQ/2014 | 2012.10-2013.09 | Guangxi | HIV-positive men and women | 142 | Mean (range): 41.8 (18-82) | 85 (59.9) | HAMD-24 ≥ 8 |
| Dwyer R/2014 | NR | Beijing | HIV-positive men and women | 50 | Mean (SD): 35 (7.3) | 42 (84) | CES-D-20 ≥ 16 |
| Hou WL/2014 | 2007.09-2010.04 | Taiwan | HIV-positive men and women | 108 | Mean (SD): 33.3 (8.5) | 366 (90.8) | BDI-II ≥ 14 |
| Liu HJ/2014 | 2011 | Guangdong | HIV-positive men and women | 148 | Mean (range): 58.4 (50-80) | 103 (69.6) | CES-D-10 ≥ 10 |
| Liu Y/2014 | 2012.06-2012.12 | Hunan | HIV-positive men and women | 262 | Mean (SD): 32.8 (9.5) | 207 (79.0) | PHQ-9 ≥ 5 |
| Peng L/2014 | 2012.08-2012.09 | Guangdong | HIV-positive men and women | 443 | Mean (range): 34 (13-67) | 303 (68.4) | HADS-D ≥ 8 |
| Qiu YY/2014 | 2013.03-2014.02 | Hunan | HIV-positive men and women | 370 | Mean (SD): 32.8 (10.8) | 341 (92.2) | PHQ-9 ≥ 10 |
| Shi K/2014 | 2013 | Beijing, Zhejiang, Hunan, Yunnan | HIV-positive men and women | 120 | Median: 38.5 | 72 (60) | Zung SDS ≥ 53 |
| Sun W/2014 | 2010.10-2011.04 | Liaoning | HIV-positive men and women | 772 | Mean (SD): 37.4 (11.2) | 691 (89.5) | CES-D-20 ≥ 16 |
| Wang HH/2014 | 2009.07-2010.07 | Hunan | HIV-positive men and women | 496 | Mean (range): 38 (20-71) | 342 (69) | CES-D-20 ≥ 16 |
| Yang GL/2014 | 2013.01-2013.07 | Hunan | HIV-positive men and women | 190 | Mean (SD): 33.7 (10.3) | 149 (78.4) | PHQ-9 ≥ 5 |
| Yao HJ/2014 | NR | Shanghai | HIV-positive men and women | 136 | Mean (range): 37 (20-69) | 131 (96.3) | Zung SDS ≥ 53 |
| Zhou G/2014 | 2012.05-2013.01 | Yunnan | HIV-positive men and women | 356 | Mean (SD): 38.1 (8.3) | 203 (57) | HADS-D ≥ 8 |
| Zhou ZH/2014 | 2012.03-2013.02 | Jiangsu | HIV-positive men and women | 41 | Mean (range): 43.2 (24-79) | 33 (80.5) | SCL-90 ≥ 2 |
| Liu L/2013 | 2010.12-2011.04 | Liaoning | HIV-positive men and women | 320 | Mean (SD): 36.9 (9.8) | 298 (93.1) | CES-D-20 ≥ 16 |
| Su XY/2013 | 2007.09-2008.01 | Guangdong, Hunan | HIV-positive men and women | 258 | Median: 35.1 | 190 (73.6) | BDI-II ≥ 14 |
| Yang YJ/2013 | 2012.05-2012.12 | Guangdong | HIV-positive men and women | 144 | Mean (SD): 36.9 (10.8) | 97 (67.4) | Zung SDS SI ≥ 0.5 |
| Bo P/2012 | 2008-2011 | Hunan | HIV-positive men and women | 145 | Median: 36.3 | 82 (56.6) | BDI ≥ 16 |
| Rao D/2012 | 2006.12-2008.04 | Beijing | HIV-positive men and women | 120 | Mean (SD): 36 (8.0) | 98 (82) | CES-D-10 ≥ 10 |
| Sun YH/2012 | 2009.05-2010.03 | Shandong | HIV-positive men and women | 36 | Mean (range): 35 (15-57) | 21 (58.3) | Zung SDS ≥ 50 |
| Yeh ML/2012 | 2008.09-2009.01 | Taiwan | HIV-positive men and women | 160 | Mean (SD): 36.8 (10.4) | 150 (93.8) | BDI-II ≥ 14 |
| Dong WY/2011 | 2010.10-2010.12 | Guangxi | HIV-positive men and women | 400 | Mean (SD): 39.3 (11.0) | 255 (63.8) | Zung SDS SI ≥ 0.5 |
| Jin C/2010 | NR | Zhejiang | HIV-positive men and women | 214 | Mean (SD): 31.3 (9.2) | 162 (75.7) | SCL-90 ≥ 2 |
| Liu TZ/2010 | -2009.05 | Shandong | HIV-positive men and women | 651 | Mean (SD): 34.37 (11.38) | 368 (56.5) | Zung SDS SI ≥ 0.5 |
| Lu L/2010 | 2008.12 | Henan | HIV-positive men and women | 144 | Median (range): 47.4 (18-73) | 92 (63.9) | Zung SDS ≥ 50 |
| Chen G/2008 | 2007.04-2007.07 | Henan | HIV-positive men and women | 192 | Mean (SD): 43.9 (9.1) | 92 (47.9) | SCL-90 ≥ 2 |
| Li BG/2008 | 2008.01-2008.04 | Yunnan | HIV-positive men and women | 95 | Mean (SD): 36.3 (6.5) | 58 (61.1) | Zung SDS SI ≥ 0.5 |
| Li J/2008 | 2007 | Anhui | HIV-positive men and women | 66 | Mean (SD): 42.1 (9.2) | 32 (48.5) | Zung SDS ≥ 53 |
| Zhu XY/2008 | 2007.03-2007.08 | Shandong | HIV-positive men and women | 105 | Mean (SD): 42.3 (8.2) | 59 (54.1) | Zung SDS SI ≥ 0.5 |
| Huang TL/2006 | 1997.10-2004.09 | Taiwan | HIV-positive men | 60 | Mean (SD): 38.1 (9.3) | 60 (100) | SCID-I |
| Jin H/2006 | 2004.03-2004.08 | Beijing, Anhui | HIV-positive men and women | 28 | Mean (SD): 35.6 (6.7) | 21 (75) | BDI ≥ 10 |
| Liao Q/2004 | NR | Sichuan | HIV-positive men and women | 71 | NR | NR | Zung SDS SI ≥ 0.5 |
| Yen CF/2004 | 1999.11-2000.05 | Taiwan | HIV-positive men | 41 | Mean (SD): 33.4 (9.3) | 41 (100) | BDI-II ≥ 11 |
| Luo SX/2017 | 2015.05-2015.12 | Chongqing | HIV-positive MSM | 400 | Median: 28 | 400 (100) | Zung SDS ≥ 50 |
| Tao J/2017 | 2013.03-2015.05 | Beijing | HIV-positive MSM | 364 | Median (IQR): 28 (25-32) | 364 (100) | HADS-D ≥ 8 |
| Li JH/2016 | 2013.07-2013.12 | Sichuan | HIV-positive MSM | 321 | NR | 321 (100) | CES-D-20 ≥ 16 |
| Li Z/2016 | 2014.04-2014.06 | Beijing | HIV-positive MSM | 266 | Mean (SD): 34.2 (9.3) | 266 (100) | CES-D-20 ≥ 16 |
| Peng BH/2016 | 2013.03-2014.08 | Hunan | HIV-positive MSM | 321 | Mean (SD): 28.4 (8.1) | 321 (100) | PHQ-9 ≥ 10 |
| Chen F/2015 | 2012.12-2013.05 | Guangdong, Chongqing, Sichuan | HIV-positive MSM | 541 | Mean (SD): 30.2 (7.7) | 541 (100) | CES-D-20 ≥ 16 |
| Wu YL/2015 | 2013.04-2013.07 | Anhui | HIV-positive MSM | 184 | Mean (SD): 31.4 (9.2) | 184 (100) | CES-D-20 ≥ 22 |
| Liu Y and Yang GL/2014 | 2012.03-2012.09 | Hunan | HIV-positive MSM | 123 | Mean (SD): 27.6 (5.5) | 123 (100) | PHQ-9 ≥ 5 |
| Sun WM/2014 | 2013.05-2013.12 | Jiangxi | HIV-positive MSM | 65 | Mean (SD): 28.3 (4.8) | 65 (100) | Zung SDS ≥ 53 |
| Wang M/2013 | 2010.08-2010.12 | Shanghai | HIV-positive MSM | 200 | Mean (SD): 36.3 (10.1) | 200 (100) | CES-D-20 ≥ 16 |
| Yang HX/2011 | 2010.01-2010.12 | Yunnan | HIV-positive pregnant women | 307 | Mean (SD): 39.3 (11.1) | 0 (0) | SCL-90 ≥ 2 |
| Wang YC/2010 | NR | Yunnan | HIV-positive pregnant women | 44 | Mean (range): 25.7 (19-37) | 0 (0) | Zung SDS SI ≥ 0.5 |
| Fang XH/2016 | NR | Anhui | HIV-TB coinfected men and women | 124 | NR | NR | Zung SDS SI ≥ 0.5 |
| Luo XY/2016 | 2014.06-2015.05 | Sichuan | HIV-TB coinfected men and women | 256 | Median: 33.6 | 192 (75.0) | BDC ≥ 53 |
| Xu MZ/2010 | 2007.09-2008.09 | Guangdong | HIV-positive IDUs | 280 | Mean (SD): 34.4 (6.3) | 258 (92.1) | HAMD-24 ≥ 8 |
| Wang HH/2008 | 2007.07-2007.09 | Hunan | HIV-positive IDUs | 111 | Mean (SD): 36.7 (6.7) | 93 (83.8) | Zung SDS ≥ 50 |
| Li Z/2015 | 2014.04-2014.08 | Beijing, Henan | HIV-positive FBPD | 239 | Median: 51.7 | 117 (49) | CES-D-20 ≥ 16 |
| Su PY/2010 | 2006.07 | Anhui | HIV-positive FBPD | 153 | Mean (SD): 40.4 (6.2) | 67 (43.8) | SCL-90 ≥ 3 |
| Ning NY/2008 | 2006.01-2006.07 | Anhui | HIV-positive FBPD | 203 | Mean (SD): 40.2 (6.4) | 244 (60.8) | BDI ≥ 10 |
| Fang GX/2007 | NR | Anhui | HIV-positive FBPD | 95 | Mean (SD): 45.2 (7.7) | 40 (42.1) | Zung SDS ≥ 54 |
| Lyu YH/2007 | 2006.12-2007.01 | Henan | HIV-positive FBPD | 202 | Median (range): 42 (29-62) | 125 (46.5) | DASS-21 ≥ 6 |
| Sun J/2006 | 2005.03-2005.04 | Hubei | HIV-positive FBPD | 156 | Mean (SD): 43.3 (7.3) | 84 (53.9) | CES-D-20 ≥ 16 |
| Lin XY/2005 | 2003.01 | NR | HIV-positive FBPD | 185 | Median (range): 38.5 (20-60) | 82 (44.6) | BDI ≥ 5 |

BDC, Burns Depression Checklist; BDI, Beck Depression Inventory; CES-D-10, 10-item Center for Epidemiological Studies Depression Scale; CES-D-20, 20-item Center for Epidemiological Studies Depression Scale; DASS-21, 21-item Depression Anxiety Stress Scale; FBPD, former blood/plasma donors; HADS-D, Hospital Anxiety and Depression Scale; HAMD-24, 24-item Hamilton Depression Rating Scale; IDUs, injected drug users; IQR, interquartile range; MSM, men who sex with men; NR, not reported; PCT V4.0, Psychological “Computerized Tomography”4.0 Vision; PHQ-9, 9-item Patient Health Questionnaire; SCID-I, Structured Clinical Interview for the fourth edition of the Diagnostic and Statistical Manual for Mental Disorders Axis I Disorders; SCL-90, 90-item Symptom Check List; SD, standard deviation; SI, severity index; Zung-SDS, Zung Self-Rating Depression Scale.
