# Supplementary material for: Prevalence of depression or depressive symptoms among people living with HIV/AIDS in China: a systematic review and meta-analysis
Source: BMC Psychiatry. 2018 May 31;18:160. doi: 10.1186/s12888-018-1741-8 (PMC5984474; doi:10.1186/s12888-018-1741-8)
Supplement: Supplementary file 4 — “Modified Newcastle-Ottawa risk of bias score for the 74 studies included in this systematic review and meta-analysis”. (DOC 190 kb) [file 12888_2018_1741_MOESM4_ESM.doc]

**Additional file 4** Modified Newcastle-Ottawa risk of bias score for the 74 studies included in this systematic review and meta-analysis

| Study | Sample representativeness | Sample size | Response rate | Ascertainment of depression* | Descriptive statistics | Total |
| --- | --- | --- | --- | --- | --- | --- |
| Hu J/2017 | 0 | 1 | 1 | 1 | 1 | 4 |
| Huang XJ/2017 | 1 | 1 | 1 | 1 | 1 | 5 |
| Liu Y/2017 | 0 | 1 | 0 | 1 | 1 | 3 |
| Luo SX/2017 | 0 | 1 | 0 | 1 | 1 | 3 |
| Mo XY/2017 | 0 | 1 | 1 | 0 | 1 | 3 |
| Rong H/2017 | 1 | 1 | 0 | 1 | 1 | 4 |
| Tao J/2017 | 1 | 1 | 1 | 1 | 1 | 5 |
| Zhao L/2017 | 1 | 1 | 0 | 1 | 1 | 4 |
| Fang XH/2016 | 1 | 1 | 1 | 1 | 0 | 4 |
| Jiang Y/2016 | 0 | 1 | 0 | 1 | 1 | 3 |
| Ju LH/2016 | 1 | 1 | 0 | 0 | 0 | 2 |
| Li C/2016 | 1 | 1 | 0 | 1 | 1 | 4 |
| Li JH/2016 | 1 | 1 | 1 | 1 | 0 | 4 |
| Li L/2016 | 0 | 1 | 0 | 0 | 1 | 2 |
| Li YL/2016 | 0 | 1 | 1 | 1 | 0 | 3 |
| Li Z/2016 | 0 | 1 | 1 | 1 | 1 | 4 |
| Luo XY/2016 | 0 | 1 | 0 | 0 | 0 | 1 |
| Peng BH/2016 | 1 | 1 | 1 | 1 | 1 | 5 |
| Sun YP/2016 | 0 | 1 | 1 | 1 | 1 | 4 |
| Zhang CH/2016 | 0 | 1 | 0 | 1 | 0 | 2 |
| Zhang X/2016 | 0 | 0 | 0 | 1 | 1 | 2 |
| Zhou J/2016 | 1 | 1 | 1 | 1 | 0 | 4 |
| Guo ZH/2015 | 0 | 1 | 1 | 1 | 1 | 4 |
| Li XH/2015 | 1 | 1 | 0 | 1 | 1 | 4 |
| Li Z/2015 | 0 | 1 | 0 | 1 | 1 | 3 |
| Sun HM/2015 | 1 | 1 | 1 | 0 | 1 | 4 |
| Chen FQ/2014 | 0 | 1 | 0 | 1 | 0 | 2 |
| Dwyer et al./2014 | 0 | 0 | 1 | 1 | 1 | 3 |
| Hou WL/2014 | 0 | 1 | 0 | 1 | 1 | 3 |
| Lau JT/2014 | 1 | 1 | 1 | 1 | 0 | 4 |
| Liu HJ/2014 | 1 | 1 | 1 | 1 | 0 | 4 |
| Liu Y and Yang GL/2014 | 1 | 1 | 1 | 1 | 1 | 5 |
| Liu Y/2014 | 1 | 1 | 1 | 1 | 0 | 4 |
| Peng L/2014 | 1 | 1 | 1 | 1 | 0 | 4 |
| Qin XJ/2014 | 0 | 1 | 0 | 1 | 1 | 3 |
| Qiu YY/2014 | 1 | 1 | 1 | 1 | 1 | 5 |
| Shi K/2014 | 1 | 1 | 0 | 0 | 0 | 2 |
| Sun W/2014 | 1 | 1 | 1 | 1 | 1 | 5 |
| Sun WM/2014 | 1 | 0 | 1 | 0 | 1 | 3 |
| Wang HH/2014 | 1 | 1 | 1 | 1 | 0 | 4 |
| Yang GL/2014 | 1 | 1 | 1 | 1 | 1 | 5 |
| Yao HJ/2014 | 0 | 1 | 0 | 0 | 0 | 1 |
| Zhou G/2014 | 1 | 1 | 1 | 1 | 1 | 5 |
| Zhou ZH/2014 | 0 | 0 | 0 | 1 | 0 | 1 |
| Jin H/2013 | 1 | 1 | 1 | 1 | 1 | 5 |
| Liu L/2013 | 1 | 1 | 1 | 1 | 1 | 5 |
| Su XY/2013 | 1 | 1 | 1 | 1 | 0 | 4 |
| Wang M/2013 | 1 | 1 | 0 | 1 | 1 | 4 |
| Yang YJ/2013 | 0 | 1 | 0 | 1 | 1 | 3 |
| Bo P/2012 | 0 | 1 | 0 | 1 | 0 | 2 |
| Rao D/2012 | 0 | 1 | 0 | 1 | 1 | 3 |
| Sun YH/2012 | 1 | 0 | 0 | 1 | 0 | 2 |
| Yeh ML/2012 | 0 | 1 | 1 | 1 | 1 | 4 |
| Dong WY/2011 | 0 | 1 | 1 | 1 | 1 | 4 |
| Yang HX/2011 | 1 | 1 | 1 | 1 | 1 | 5 |
| Jin C/2010 | 1 | 1 | 0 | 1 | 1 | 4 |
| Liu TZ/2010 | 1 | 1 | 1 | 1 | 1 | 5 |
| Lu L/2010 | 0 | 1 | 1 | 1 | 0 | 3 |
| Peng EY/2010 | 1 | 1 | 1 | 1 | 1 | 5 |
| Wang YC/2010 | 1 | 0 | 0 | 1 | 0 | 2 |
| Xu MZ/2010 | 1 | 1 | 1 | 1 | 1 | 5 |
| Ren YX/2009 | 1 | 1 | 1 | 1 | 1 | 5 |
| Chen G/2008 | 0 | 1 | 1 | 1 | 1 | 4 |
| Li BG/2008 | 0 | 0 | 0 | 1 | 1 | 2 |
| Li J/2008 | 0 | 0 | 1 | 0 | 1 | 2 |
| Wang HH/2008 | 1 | 1 | 0 | 1 | 1 | 4 |
| Zhu XY/2008 | 1 | 1 | 1 | 1 | 1 | 5 |
| Fang GX/2007 | 0 | 0 | 0 | 1 | 1 | 2 |
| Wu HY/2007 | 1 | 1 | 0 | 1 | 1 | 4 |
| Huang TL/2006 | 0 | 0 | 0 | 1 | 1 | 2 |
| Sun J/2006 | 1 | 1 | 0 | 1 | 1 | 4 |
| Lin XY/2005 | 0 | 1 | 1 | 0 | 0 | 2 |
| Liao Q/2004 | 1 | 1 | 0 | 1 | 0 | 3 |
| Yen CF/2004 | 0 | 0 | 1 | 1 | 1 | 3 |

*The sensitivity and specificity of survey instruments for diagnosing depressive disorder were shown in Supplement Table S2.
