# Supplementary material for: Prevalence of depression or depressive symptoms among people living with HIV/AIDS in China: a systematic review and meta-analysis
Source: BMC Psychiatry. 2018 May 31;18:160. doi: 10.1186/s12888-018-1741-8 (PMC5984474; doi:10.1186/s12888-018-1741-8)
Supplement: Supplementary file 5 — “Sensitivities and specificities of commonly used instruments for diagnosing depression”. (DOC 43 kb) [file 12888_2018_1741_MOESM5_ESM.doc]

**Additional file 5** Sensitivities and specificities of commonly used instruments for diagnosing depression

| Instrument | Cut off | Sensitivity | Specificity |
| --- | --- | --- | --- |
| Burns depression checklist (BDC) | ≥ 53 | - | - |
| Beck Depression Inventory (BDI) | ≥ 5 | - | - |
| Beck Depression Inventory (BDI) | ≥10 | 100 | 93.75 |
| Beck Depression Inventory (BDI) | ≥16 | 79.0 | 91.0 |
| Beck Depression Inventory-II (BDI-II) | ≥11 | 95.9 (92-100) | 71 (66-80) |
| Beck Depression Inventory-II (BDI-II) | ≥14 | 88 | 84 |
| Center for Epidemiologic Studies Depression Scale-10 items (CESD-10) | ≥10 | 82 | 98.4 |
| Center for Epidemiologic Studies Depression Scale-20 items (CESD-20) | ≥16 | 87 | 70 |
| Center for Epidemiologic Studies Depression Scale-20 items (CESD-20) | ≥17 | 86.6 | 72.2 |
| Center for Epidemiologic Studies Depression Scale-20 items (CESD-20) | ≥20 | 83 | 78 |
| Center for Epidemiologic Studies Depression Scale-20 items (CESD-20) | ≥22 | 79 | 80 |
| Depression Anxiety Stress Scale-21 (DASS-21) | ≥ 6 | - | - |
| Psychological“ Computerized Tomography”4.0 Vision (PCT V4.0) | - | 97.0 | 96.8 |
| Symptom Check List-90 (SCL-90) | ≥ 2 | 72.9 | 90.3 |
| Symptom Check List-90 (SCL-90) | ≥ 3 | - | - |
| Patient Health Questionnaire-9 (PHQ-9) | ≥ 5 | 92.3 | 70.4 |
| Patient Health Questionnaire-9 (PHQ-9) | ≥ 10 | 81.3 | 85.3 |
| Hamilton Depression Rating Scale-24 items (HAMD-24) | ≥ 8 | 91 | 72 |
| Hospital Anxiety and Depression Scale (HADS-D) | ≥ 8 | 80 | 88 |
| Zung Self-Rating Depression Scale (SDS) | ≥ 50 | 86 (73-100) | 76 (57-95) |
| Zung Self-Rating Depression Scale (SDS) | ≥ 53 | - | - |
| Zung Self-Rating Depression Scale (SDS) | ≥ 54 | 92.3 | 87.5 |
