# Supplementary material for: Prevalence of depression or depressive symptoms among people living with HIV/AIDS in China: a systematic review and meta-analysis
Source: BMC Psychiatry. 2018 May 31;18:160. doi: 10.1186/s12888-018-1741-8 (PMC5984474; doi:10.1186/s12888-018-1741-8)
Supplement: Supplementary file 6 — “Sensitivity analysis of the prevalence of depression or depressive symptoms among people living with HIV/AIDS in China”. (PDF 157 kb) [file 12888_2018_1741_MOESM6_ESM.pdf]

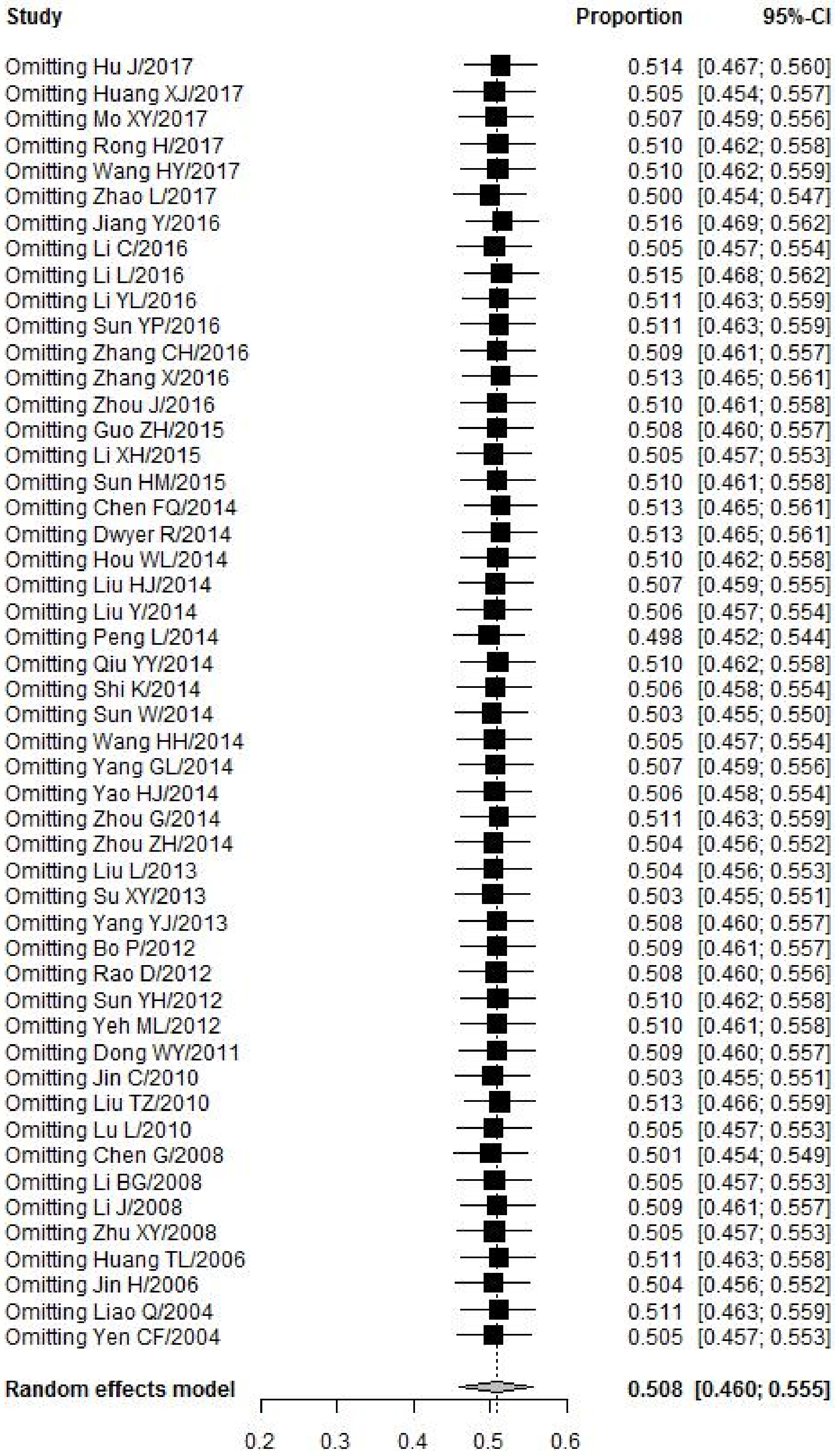

Additional file 6 Sensitivity analysis of the prevalence of depression or depressive symptoms among people living with HIV/AIDS in China.
