# Supplementary material for: Prevalence of depression or depressive symptoms among people living with HIV/AIDS in China: a systematic review and meta-analysis
Source: BMC Psychiatry. 2018 May 31;18:160. doi: 10.1186/s12888-018-1741-8 (PMC5984474; doi:10.1186/s12888-018-1741-8)
Supplement: Supplementary file 7 — “Within-instrument heterogeneity analyses of studies reporting on the prevalence of depression or depressive symptoms among people living with HIV/AIDS in China: stratified meta-analyses and meta-regression analyses”. (DOC 200 kb) [file 12888_2018_1741_MOESM7_ESM.doc]

**Additional file 7** Within-instrument heterogeneity analyses of studies reporting on the prevalence of depression or depressive symptoms among people living with HIV/AIDS in China: stratified meta-analyses and meta-regression analyses

**Supplementary Table 1 Stratified Meta-Analyses**

| Characteristics | No. of Studies | No. Depressed | Total No. | Prevalence, % (95%CI) | I^2^ (%) | *P* value for heterogeneity | Test for subgroup differences | |
| --- | --- | --- | --- | --- | --- | --- | --- | --- |
|  |  |  |  |  |  |  | Q (df) | *P* value |
| **Zung Self-Rating Depression Scale Score ≥ 50** |  |  |  |  |  |  |  |  |
| Geographic location |  |  |  |  |  |  | 0.3 | 0.961 |
| Central China | 3 | 380 | 1,002 | 42.4 (24.9, 62.1) | 96.9 | < 0.001 |  |  |
| East China | 2 | 266 | 765 | 42.9 (21.7, 67.1) | 95.2 | < 0.001 |  |  |
| South China | 2 | 254 | 544 | 46.7 (42.5, 50.9) | 0 | 0.731 |  |  |
| Southwest | 2 | 85 | 166 | 49.3 (24.0, 75.0) | 91.9 | < 0.001 |  |  |
| Sample source |  |  |  |  |  |  | 0.2 | 0.630 |
| Community-based | 5 | 360 | 980 | 42.4 (29.5, 56.4) | 92.1 | < 0.001 |  |  |
| Hospital-based | 5 | 625 | 1,497 | 47.1 (34.5, 60.0) | 95.5 | < 0.001 |  |  |
| Total NOS score |  |  |  |  |  |  | 0.2 | 0.630 |
| < 3 points | 2 | 73 | 131 | 50.5 (25.6, 75.1) | 86.5 | 0.006 |  |  |
| ≥ 3 points | 8 | 912 | 2,346 | 43.4 (34.5, 52.8) | 94.4 | < 0.001 |  |  |
| **Symptom Checklist Score-90 ≥ 2** |  |  |  |  |  |  |  |  |
| Geographic location |  |  |  |  |  |  | 0.3 | 0.571 |
| East China | 3 | 228 | 365 | 62.4 (40.3, 80.3) | 92.7 | < 0.001 |  |  |
| Others^a^ | 4 | 304 | 502 | 53.4 (31.5, 74.0) | 95.3 | < 0.001 |  |  |
| Sample source |  |  |  |  |  |  | 0.2 | 0.652 |
| Community-based | 5 | 488 | 769 | 60.7 (45.7, 73.9) | 93.5 | < 0.001 |  |  |
| Hospital-based | 2 | 44 | 98 | 48.3 (10.4, 88.3) | 95.2 | < 0.001 |  |  |
| Total NOS score |  |  |  |  |  |  | 1.4 | 0.230 |
| < 3 points | 3 | 89 | 201 | 46.5 (23.8, 70.8) | 90.3 | < 0.001 |  |  |
| ≥ 3 points | 4 | 443 | 666 | 64.6 (48.9, 77.7) | 93.2 | < 0.001 |  |  |
| **Zung Self-Rating Depression Scale Score ≥ 53** |  |  |  |  |  |  |  |  |
| Geographic location |  |  |  |  |  |  | 2.5 | 0.115 |
| East China | 4 | 271 | 666 | 40.8 (26.0, 57.6) | 93.6 | < 0.001 |  |  |
| Others^b^ | 2 | 241 | 441 | 55.4 (48.4, 62.3) | 47.3 | 0.166 |  |  |
| Sample source |  |  |  |  |  |  | 0.3 | 0.605 |
| Community-based | 2 | 155 | 364 | 42.6 (37.6, 0.477) | 0 | 0.805 |  |  |
| Hospital-based | 4 | 357 | 743 | 47.4 (30.6, 64.9) | 95.1 | < 0.001 |  |  |
| Total NOS score |  |  |  |  |  |  | 0.1 | 0.851 |
| < 3 points | 4 | 217 | 488 | 45.2 (25.9, 66.0) | 94.9 | < 0.001 |  |  |
| ≥ 3 points | 2 | 295 | 619 | 47.5 (37.5, 57.6) | 84.9 | 0.010 |  |  |
| **20-Item Center for Epidemiological Studies Depression Scale Score ≥ 16** |  |  |  |  |  |  |  |  |
| Geographic location |  |  |  |  |  |  | 29.7 | < 0.001 |
| Central China | 2 | 384 | 610 | 62.9 (59.0, 66.7) | 0 | 0.487 |  |  |
| North China | 1 | 12 | 50 | 24.0 (14.2, 37.7) | - | - |  |  |
| Northeast | 2 | 778 | 1,092 | 70.0 (63.0, 76.2) | 80.3 | 0.024 |  |  |
| Sample source |  |  |  |  |  |  | 3.5 | 0.063 |
| Community-based | 2 | 775 | 1,092 | 70.0 (63.0, 76.2) | 80.3 | 0.024 |  |  |
| Hospital-based | 3 | 396 | 660 | 51.7 (33.4, 69.6) | 91.9 | < 0.001 |  |  |
| Total NOS score |  |  |  |  |  |  | - | - |
| < 3 points | - | - | - | - | - | - |  |  |
| ≥ 3 points | 5 | 1,172 | 1,752 | 60.9 (51.2, 69.8) | 91.9 | < 0.001 |  |  |

a: including one study from Central China, North China, Northeast and Northwest; b: including one study from South China and Cross-region

Supplementary Table 2 Meta-regression Analyses

| Study characteristics | Estimate | SE | *P* | 95%CI | Moderator analysis^a^ | |
| --- | --- | --- | --- | --- | --- | --- |
|  |  |  |  |  | DLT (df) | *P* |
| **Zung Self-Rating Depression Scale Score ≥ 50** |  |  |  |  |  |  |
| Year of survey | -0.152 | 0.043 | < 0.001 | -0.236, -0.067 | 12.457 (1) | < 0.001 |
| Sample size | -0.002 | 0.001 | 0.029 | -0.003, -0.001 | 4.789 (1) | 0.029 |
| Age (years) | 0.113 | 0.032 | < 0.001 | 0.051, 0.175 | 12.685 (1) | < 0.001 |
| Male (proportion, %) | -0.020 | 0.019 | 0.282 | -0.058, 0.017 | 1.158 (1) | 0.282 |
| HIV-positive individuals with ART (proportion, %) | 0.003 | 0.006 | 0.577 | -0.009, 0.016 | 0.310 (1) | 0.577 |
| **Symptom Checklist Score-90 ≥ 2** |  |  |  |  |  |  |
| Year of survey | -0.210 | 0.080 | 0.009 | -0.366, -0.053 | 6.896 (1) | 0.009 |
| Sample size | 0.008 | 0.004 | 0.030 | 0.001, 0.015 | 4.692 (1) | 0.030 |
| Age (years) | 0.055 | 0.050 | 0.273 | -0.043, 0.152 | 1.202 (1) | 0.273 |
| Male (proportion, %) | -0.021 | 0.017 | 0.200 | -0.054, 0.011 | 1.643 (1) | 0.200 |
| HIV-positive individuals with ART (proportion, %) | 0.005 | 0.013 | 0.714 | -0.021, 0.030 | 0.134 (1) | 0.714 |
| **Zung Self-Rating Depression Scale Score ≥ 53** |  |  |  |  |  |  |
| Year of survey | -0.295 | 0.291 | 0.311 | -0.866, 0.276 | 1.025 (1) | 0.311 |
| Sample size | -0.001 | 0.003 | 0.931 | -0.006, 0.005 | 0.008 (1) | 0.931 |
| Age (years) | 0.063 | 0.049 | 0.198 | -0.033, 0.160 | 1.657 (1) | 0.198 |
| Male (proportion, %) | -0.011 | 0.013 | 0.392 | -0.038, 0.015 | 0.734 (1) | 0.392 |
| HIV-positive individuals with ART (proportion, %) | -0.021 | 0.034 | 0.549 | -0.088, 0.047 | 0.360 (1) | 0.549 |
| **20-Item Center for Epidemiological Studies Depression Scale Score ≥ 16** |  |  |  |  |  |  |
| Year of survey | 0.303 | 0.241 | 0.210 | -0.170, 0.775 | 1.573 (1) | 0.210 |
| Sample size | 0.002 | 0.001 | 0.021 | 0.001, 0.003 | 5.311 (1) | 0.021 |
| Age (years) | 0.416 | 0.175 | 0.017 | 0.073, 0.758 | 5.658 (1) | 0.017 |
| Male (proportion, %) | 0.003 | 0.024 | 0.885 | -0.043, 0.050 | 0.021 (1) | 0.885 |
| HIV-positive individuals with ART (proportion, %) | -0.004 | 0.010 | 0.687 | -0.023, 0.015 | 0.162 (1) | 0.687 |

DLT, DerSimonian-Laird test; NOS, Newcastle-Ottawa Scare; SE, standard error.

aCompares model with no variables versus model with each variable separately via DerSimonian-Laird test to test the effect of each variable on the between-study heterogeneity and indicates the proportion of residual heterogeneity in the simplified model accounting for the heterogeneity in the full model.
